# QingXiaoWuWei decoction alleviates methicillin-resistant *Staphylococcus aureus*-induced pneumonia in mice by regulating metabolic remodeling and macrophage gene expression network via the microbiota-short-chain fatty acids axis

**DOI:** 10.1128/spectrum.00344-23

**Published:** 2023-10-12

**Authors:** Jun Li, Qian Zhang, Xue Li, Jing Liu, Fang Wang, Wei Zhang, Xingyue Liu, Tiewei Li, Shiqi Wang, Yuqi Wang, Xinyu Zhang, Yukun Meng, Yuheng Ma, Huanyun Wang

**Affiliations:** 1 College of Pharmacy, Inner Mongolia Medical University, Hohhot, China; 2 First Clinical Medical College, Inner Mongolia Medical University, Hohhot, China; 3 Zhengzhou Key Laboratory of Children’s Infection and Immunity, Children’s Hospital Affiliated to Zhengzhou University, Henan Children’s Hospital, Zhengzhou, China; University of Saskatchewan, Saskatoon, Saskatchewan, Canada

**Keywords:** QingXiaoWuWei decoction, MRSA-induced pneumonia, microbiota-SCFAs axis, multi-omics approaches, metabolic remodeling, macrophage gene expression network

## Abstract

QingXiaoWuWei decoction (QXWWD) exerts a prominent therapeutic effect on the methicillin-resistant *Staphylococcus aureus* (MRSA)-induced pneumonia model in mice; however, its pharmacological mechanisms remain unclear. This study aimed to investigate the underlying pharmacological mechanisms of QXWWD in MRSA-induced pneumonia. In the present study, 62 compounds were identified using high-resolution mass spectrometry. Network analysis, leveraging mass spectrometry, pinpointed the infection-linked, immunity-associated, and inflammation-related pathways as predominant targets. QXWWD significantly alleviated MRSA-induced pneumonia in mice and decreased the levels of pro-inflammatory cytokines and chemokines. 16S ribosomal RNA (16S rRNA) sequencing revealed that QXWWD regulated gut microbiota composition in mice with MRSA-induced pneumonia, which correlated with the enrichment of certain short-chain fatty acids (SCFAs)-producing strains. Further analysis with targeted metabolomics confirmed that the acetic, propionic, and butyric acid levels in the mice’s serum were elevated significantly after QXWWD treatment. The fecal microbiota transplantation experiment suggested that gut microbiota from QXWWD-treated mice and SCFAs treatment may alleviate MRSA-induced pneumonia. Additionally, the untargeted metabolomic analysis further demonstrated that metabolic remodeling is significantly regulated by the QXWWD, particularly by the enhancement of the citrate cycle. In the case of QXWWD treatment, global transcriptome profiling revealed that genes, such as *NLRP12* and *CYP1A1*, associated with macrophage antibacterial and immune activity, were downregulated. The results revealed that QXWWD regulated metabolic remodeling and macrophage gene expression network via the microbiota-SCFAs axis and thus alleviated MRSA-induced pneumonia in mice.

**IMPORTANCE:**

Methicillin-resistant *Staphylococcus aureus* (MRSA) colonizes the upper respiratory airways and is resistant to antibiotics. MRSA is a frequently acquired infection in hospital and community settings, including cases of MRSA-induced pneumonia. Multidrug-resistant *Staphylococcus aureus* and the limited efficacy of antibiotics necessitate alternative strategies for preventing or treating the infection. QingXiaoWuWei decoction (QXWWD) protects against both gut microbiota dysbiosis and MRSA-induced pneumonia. Furthermore, the QXWWD-regulated metabolic remodeling and macrophage gene expression network contribute to its protective effects through the microbiota-short-chain fatty acid axis. The results of this study suggest that QXWWD and its pharmacodynamic compounds might have the potential to prevent and treat pulmonary infections, especially those caused by multidrug-resistant organisms. Our study provides a theoretical basis for the future treatment of pulmonary infectious diseases by manipulating gut microbiota and their metabolites via traditional Chinese medicine.

## INTRODUCTION

The gastrointestinal tract is hosted in a microbial environment that is both complicated and rich in variety. It is also known as the gut microbiota and is dominated by the *Bacteroidetes*, *Firmicutes*, *Proteobacteria*, and *Actinobacteria* (1). Previous research has highlighted the significance of gut microbiota and its crucial role in maintaining human health (2). The tightly regulated interaction between the microbiota and the host affects the immune system’s priming, instruction, and development (3). Some recent studies have also revealed the important role of that interaction in infection control (4). The microbial metabolites, related molecular patterns, and interactions among microorganisms, progenitors, and mature immune cells, play a critical role in regulating gut microbiota immunological responses (5).

The gut microbiota has recently been shown to have a protective function in the host’s defense against infections, particularly regarding respiratory immunity. In reality, the idea of interaction between the pulmonary immune system and the gut microbiota is now generally acknowledged and backed up by new evidence and findings. For example, mice with gut microbiota dysbiosis had greater bacterial spread and suffered severe damage induced by the host’s inflammatory response to *Streptococcus pneumoniae* infections (6). The current science has discovered molecular processes supporting such a link, which is now known as the “lung-gut axis”. The mounting evidence also shows bidirectional communication between the microbes of the “lung” and “gut” (7). These findings have motivated researchers to examine ways to maintain or induce gut microbiota homeostasis as a novel possible therapeutic approach for infectious lung diseases.

Methicillin-resistant *Staphylococcus aureus* (MRSA) infection is one of the leading causes of death in pulmonary infections caused by *Staphylococcus aureus* (8). Alternative host-directed therapeutic techniques are being produced to reduce drug-resistant strains, inflammation, damaged tissue, disease mortality, and severity (9). In addition to their primary functions, these agents can modulate the immune response through the regulation of cytokine production and by counteracting the activity of various immune cells, including neutrophils, which are quintessential to *Staphylococcus aureus* infections. This immunomodulatory capability adds another layer of complexity to these agents' multifaceted roles in biological systems. Besides, early processes during *Staphylococcal* infection include the innate immune response mediated by macrophages, which activates inflammatory signaling, produces pro-inflammatory cytokines, and generates reactive oxygen species (ROS). The excess ROS causes oxidative stress, mitochondrial damage, and inflammation (10). Thus, pharmaceutical agents have the potential to augment the antibacterial prowess of phagocytic cells and impede inflammatory reactions through various mechanisms, such as the modulation of intestinal microbiota and metabolites.

Traditional Chinese medicine (TCM) highlights holistic concepts such as mutual relationships of Yin-Yang, which is consistent with the underlying meaning of host-directed therapeutic procedures. The TCM has been shown in multiple investigations to be able to treat intestinal microecological abnormalities induced by pathogen infections (11). The phytochemicals like apigenin, baicalin, berberine (12), and sulforaphane, for example, have been found to help restore flora balance in the normal gut by increasing the probiotic organisms in the gut following pathogen infection (13). The modulation of short-chain fatty acid (SCFA) levels was connected to one probable mechanism associated with the favorable benefits of baicalin drugs for protecting rats from gut microbiota imbalance (14). Furthermore, the polysaccharides that are TCM-based have been demonstrated to enhance the synthesis of SCFAs and increase the number of some beneficial gut bacteria by immune-protective action, particularly the genera *Ruminococcus* and *Prevotella*, which belong to the *Firmicutes* and *Bacteroidetes* phyla, respectively (15).

The QingXiaoWuWei decoction (QXWWD) is an effective clinical formula for anti-inflammatory and antibacterial purposes. It is composed of *Sophorae flavescentis radix* (Kushen in Chinese), *Artemisia Argyi Folium* (Aiye in Chinese), *Fructus cnidii* (Shechuangzi in Chinese), *Rhei Radix et Rhizoma* (Dahuang in Chinese), and *Borneolum* (Bingpian in Chinese) (16). Detailed information about the herbal composition and medicinal parts of QXWWD is shown in Table 1, the plant names were checked with http://www.theplantlist.org on 16 December 2022 (17). Previous and recent studies on QXWWD (16) have shown that it significantly alleviates the pathological manifestations of lung infection and reduces the levels of pro-inflammatory cytokines and chemokines in mice with MRSA-induced pneumonia, thereby mitigating lung injury. This may primarily be achieved through its positive impact on the gut microbiota and its metabolites, SCFAs. This study aimed to investigate the pharmacological mechanism(s) responsible for the beneficial effect QXWWD treatment has on MRSA-induced pneumonia. In this study, we thoroughly examined the chemical components of QXWWD that were associated with infection, inflammation, and immune targets. Our findings show QXWWD treatment increased microbiota-derived SCFAs, which promotes intestinal health in a mouse model. Additionally, the untargeted metabolomic analysis further demonstrated that metabolic remodeling is significantly regulated by QXWWD, particularly by the enhancement of the citrate cycle (TCA cycle). In QXWWD treatment, the global transcriptome profiling revealed that genes associated with macrophage antibacterial and immune activity were differentially expressed. These findings describe the mechanism by which QXWWD treatment alleviates pathology associated with MRSA-induced pneumonia, pointing to QXWWD’s potential therapeutic role in treating refractory bacterial pneumonia.

**TABLE 1:** Detailed information on herbs in QXWWD

Chinese name	Latin name	Medical plants name	Parts used	Proportion (g)
Kushen	*Sophorae flavescentis radix*	*Sophora flavescens* Aiton	Dried roots	3
Aiye	*Artemisia Argyi Folium*	*Artemisia argyi* H.Lév. & Vaniot	Dried leaves	3
Shechuangzi	*Fructus cnidii*	*Cnidium monnieri* (L.) Cusson	Dried and ripe fructus	3
Dahuang	*Rhei Radix et Rhizoma*	*Rheum palmatum* L.	Dried roots and rhizomes	3
Bingpian	*Borneolum*	*Cinnamomum camphora* (L.) J. Presl	Extraction of branches and leaves	0.15

## RESULTS

### Composition analysis of QXWWD

**Fig 1:**
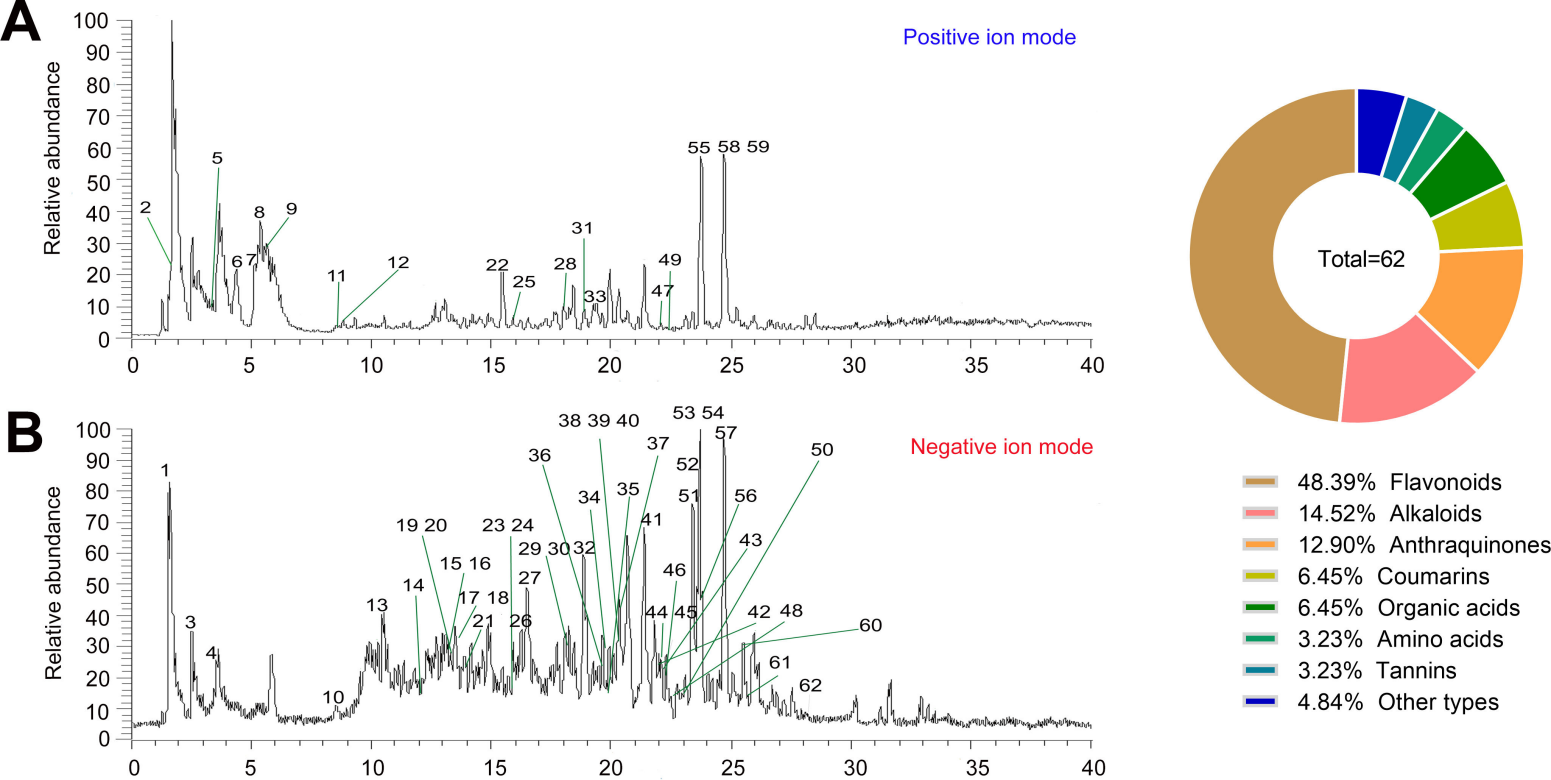
QXWWD base peak by mass spectrum with (A) positive and (B) negative modes.

Despite positive results from treating MRSA-induced pneumonia in mouse models with QXWWD, its chemical composition remains unknown, thus impeding further exploration of its pharmacological mechanisms. The chemical composition of QXWWD was initially investigated using high-resolution mass spectrometry. After evaluating the raw data, 62 significant ion peaks were identified (Fig. 1). The candidate structures were identified, derived from the public and literature databases, and validated using MS^2^ fragmentations. There were 30 flavonoids, 9 alkaloids, 8 anthraquinones, 4 each coumarin and organic acids, 2 each amino acids and tannins, and 1 each phenol, terpene, and stilbene (Table S1).

### High-resolution mass spectrometry-based network pharmacology

**Fig 2:**
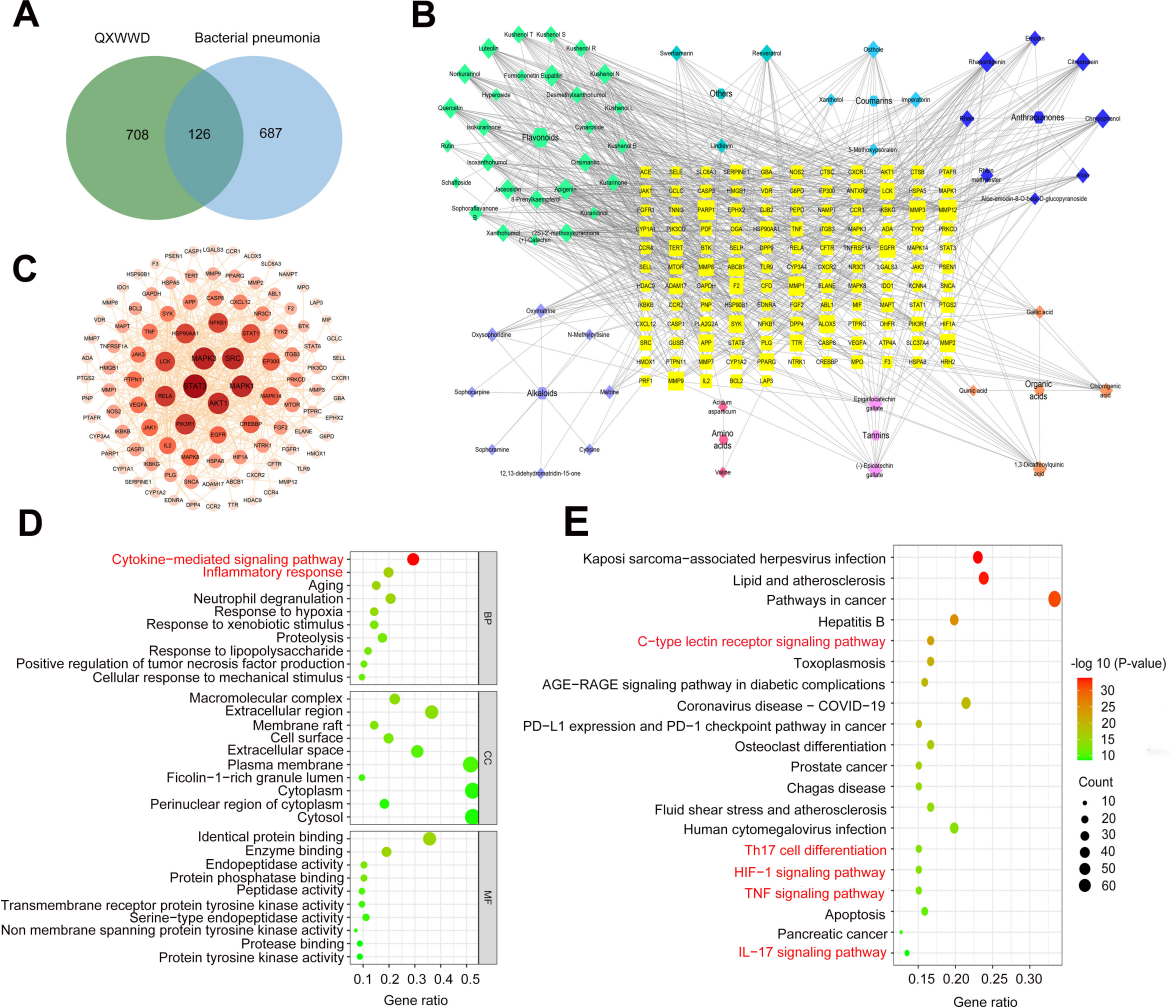
QXWWD network pharmacology analysis by high-resolution mass spectrometry. (A) Venn diagram of 813 disease targets and 834 mass high-resolution spectrometry-based QXWWD compounds yielded 126 core targets. (B) High-resolution mass spectrometry-based compound-target network. Nodes with different colors represent different compound classifications. Nodes indicate tested active components and targets, whereas connections reflect biological interactions. The "Degree" in a PPI network (C) refers to the number of node connections reflecting node interaction. The relevance of core targets was measured by "Degree". (D) Gene Ontology (GO) enrichment analysis included BP (biological processes), MF (molecular functions), and CC (cellular components). (E) KEGG pathway analysis included the Th17 cell differentiation, HIF-1 signaling pathway, TNF signaling pathway, and IL-17 signaling pathways. For statistical significance, the *P* value of enrichment analysis is <0.05.

The overlapping genes were used to construct a compound-target network to further explore the therapeutic effect of QXWWD in the MRSA-induced pneumonia model. In total, 813 disease targets and 834 compounds identified in QXWWD through high-resolution mass spectrometry resulted in 126 core targets (Fig. 2A). The core targets were entered into the visualization network program, Cytoscape 3.8.0, to create the compound-target (Fig. 2B) and protein-protein interaction (PPI) (Fig. 2C) networks. The David database was then used for functional enrichment analyses on these targets. A total of 656 biological event terminologies (Table S2) were selected, including inflammatory response and cytokine-mediated signaling pathways (Fig. 2D). The primary effects associated with the QXWWD were then categorized by the KEGG (Kyoto Encyclopedia of Genes and Genomes) pathway enrichment method, and the results are shown in Table S3. The cell differentiation of the T helper cell 17 (Th17), hypoxia inducible factor-1 (HIF-1), tumor necrosis factor (TNF), interleukin-17 (IL-17), and C-type lectin receptor signaling pathways, all of which are important in stimulating immune responses against microbial infections, were among the top QXWWD-related pathways (Fig. 2E). The findings from network pharmacology based on high-resolution mass spectrometry clearly showed that QXWWD plays a regulatory function in pathogen invasion immune response.

### QXWWD significantly alleviates MRSA-induced pneumonia in mice

**Fig 3:**
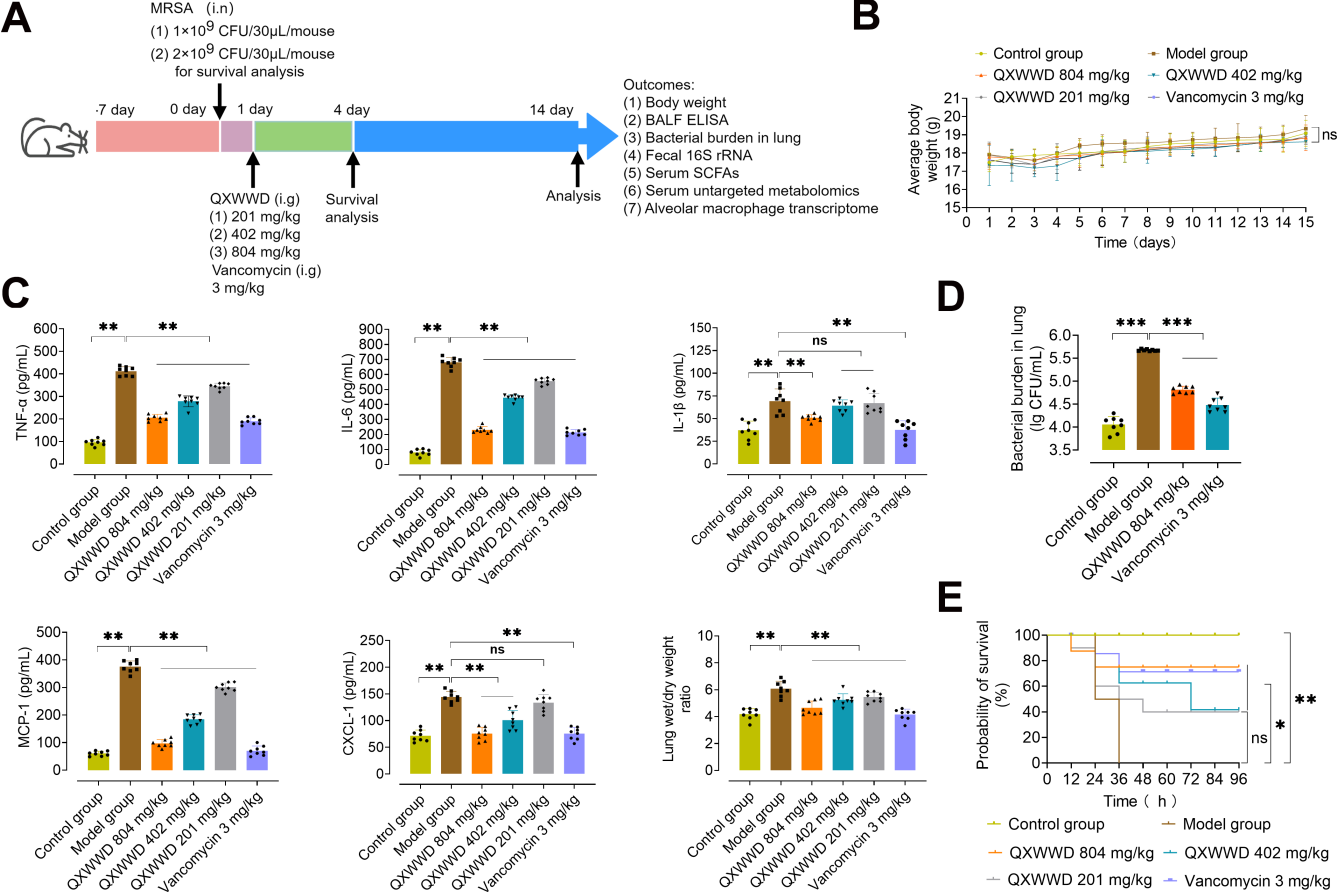
QXWWD alleviates MRSA-induced pneumonia in mice. (A) Scheme of MRSA infection and QXWWD administration. (B) Body weight change within 14 days of dosing. (C) Effects of QXWWD (high dose: 804 mg/kg, medium dose: 402 mg/kg, low dose: 201 mg/kg) and vancomycin (3 mg/kg) on inflammatory cytokines and chemokines secretion in bronchoalveolar lavage fluid (BALF) and lung *W*/*D* ratio induced by MRSA (1 × 10^9^ CFU/30 µL/mouse). (D) Pulmonary bacterial burdens in QXWWD (804 mg/kg) or vancomycin (3 mg/kg)-treated and untreated controls intranasal challenge with 1 × 10^9^ CFU/30 µL/mouse of MRSA. (E) Mice were instilled with QXWWD (201, 402, 804 mg/kg), vancomycin, or vehicle after MRSA (2 × 10^9^ CFU/30 µL/mouse) infection. Mantel-Cox survival plots were depicted, and the 96-h survival rates were analyzed. A total of eight animals were considered as a sample from each of the groups and were examined. The results are represented as mean ± standard deviation (SD) with one-way analysis of variance (ANOVA) followed by the least significant difference (LSD) multiple comparisons test; ^*^ *P* < 0.05, ^**^ *P* < 0.01, and ^***^ *P* < 0.001 are considered statistically significant.

**Fig 4:**
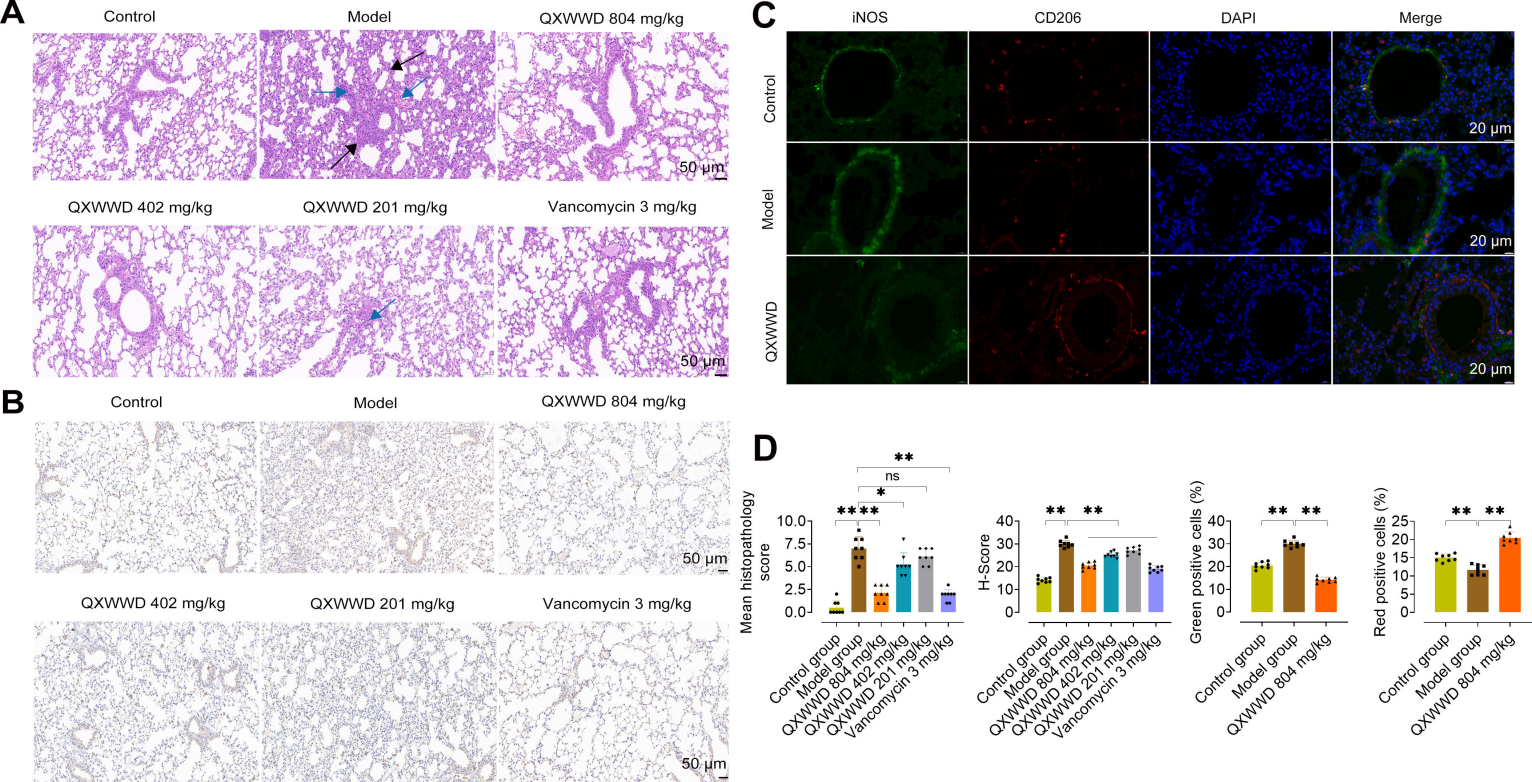
QXWWD inhibits lung inflammation and inflammatory cell infiltration in mice. Lung tissue hematoxylin-eosin (H&E) staining (A) of control, MRSA-induced pneumonia model, QXWWD 804 mg/kg, QXWWD 402 mg/kg, QXWWD 201 mg/kg, and vancomycin 3 mg/kg groups, scale bar = 50 µm. (B) Anti-F4/80 immunohistochemistry of the lung tissue of control, MRSA-induced pneumonia model, QXWWD 804 mg/kg, QXWWD 402 mg/kg, QXWWD 201 mg/kg, and vancomycin 3 mg/kg groups, scale bar = 50 µm. (C) Immunofluorescent analysis of *iNOS* (green) and *CD206* (red) in mouse lung sections. Nuclei were stained with 4',6-diamidino-2-phenylindole (DAPI) (blue), scale bar = 20 µm. (D) Quantification of mice lungs means histopathology score, H-score, and positive cell ratio (green and red fluorescence). An image of a single section was obtained from each mouse, and eight mice were in each group. Data are mean ± SD. ^*^ *P* < 0.05 and ^**^ *P* < 0.01. Statistical analysis was performed using the LSD multiple comparisons test.

QXWWD has a significant inhibitory effect on MRSA, and the minimum inhibitory concentration was approximately 7.81 µg/mL, according to our previous research (16). The function of QXWWD in the MRSA-induced mice pneumonia model to confirm the expected involvement of immune modulation of QXWWD was evaluated by experimental animal design (Fig. 3A). There was a non-significant statistical difference in the body weight between control- and drug-administrated mice (Fig. 3B), signifying that there were no adverse effects of QXWWD on the drinking and eating habits of the animals. The administration of 1 × 10^9^ CFU/30 µL/mouse of MRSA was sufficient to induce pneumonia in mice, as indicated by the significant increase in levels of pro-inflammatory cytokines and chemokines, such as interleukin-6 (IL-6), tumor necrosis factor-α (TNF-α), monocyte chemoattractant protein-1 (MCP-1), chemokine ligand-1 (CXCl-1), and interleukin-1β (IL-1β), along with lung colony-forming unit (CFU) and the wet-to-dry weight (*W*/*D*) ratio. Along with pro-inflammatory cytokines and chemokines and the wet-to-dry weight (*W*/*D*) ratio, which were all significantly reduced upon QXWWD or vancomycin treatment (Fig. 3C). Additionally, bacterial burden in the mouse lung following QXWWD treatment was significantly reduced compared to our MRSA-induce MRSA-induced pneumonia model (Fig. 3D). These results revealed that QXWWD treatment for 14 days significantly inhibited MRSA-induced pneumonia. The survival rates of mice induced by the MRSA lethal challenge (2 × 10^9^ CFU/30 µL/mouse) were evaluated in vancomycin (3 mg/kg) and QXWWD (804 mg/kg, 402 mg/kg, and 201 mg/kg)-treated mice as compared to the control group. The vancomycin (3 mg/kg) and QXWWD (804 mg/kg and 402 mg/kg) significantly reduced the 96-h mortality rates in MRSA-induced pneumonia in mice (Fig. 3E). As shown in Fig. 4A, the alveolar septa of MRSA-stimulated animals were substantially dilated (black arrow), with severe inflammatory cell infiltration (blue arrow), as compared to the undamaged control group. A protective effect of QXWWD on MRSA-induced pneumonia was shown by the fact that pulmonary pathological changes were significantly reduced in mice given either QXWWD or vancomycin. As shown in Fig. 4B, compared with the control and model groups, as expected, the immunohistochemical results showed that QXWWD and vancomycin administration significantly decreased the macrophage biomarker F4/80 in mice lung tissue. We also analyzed the immunological changes in the lung tissue to determine the effects of QXWWD therapy on lung tissue macrophages. Immunofluorescence results (Fig. 4C) showed that the production of functional *iNOS* among M1 macrophages also increased after MRSA infection. In addition, we also observed the upregulation of *CD206* among M2 macrophages after QXWWD treatment. These results suggest that QXWWD may mediate the effects of the anti-inflammation function of macrophages. The pathological images were further quantified for the mean histopathology score, H-Score (histochemistry score), and positive cells ratio (green and red fluorescence) (Fig. 4D) . The results indicated these pathological phenotypes were largely resolved in the mice with QXWWD treatment. The findings in the present study indicate that therapy with QXWWD offers a significant degree of protection against pneumonia brought on by MRSA induction.

### QXWWD regulates the composition of the gut microbiota in mice with MRSA-induced pneumonia

**Fig 5:**
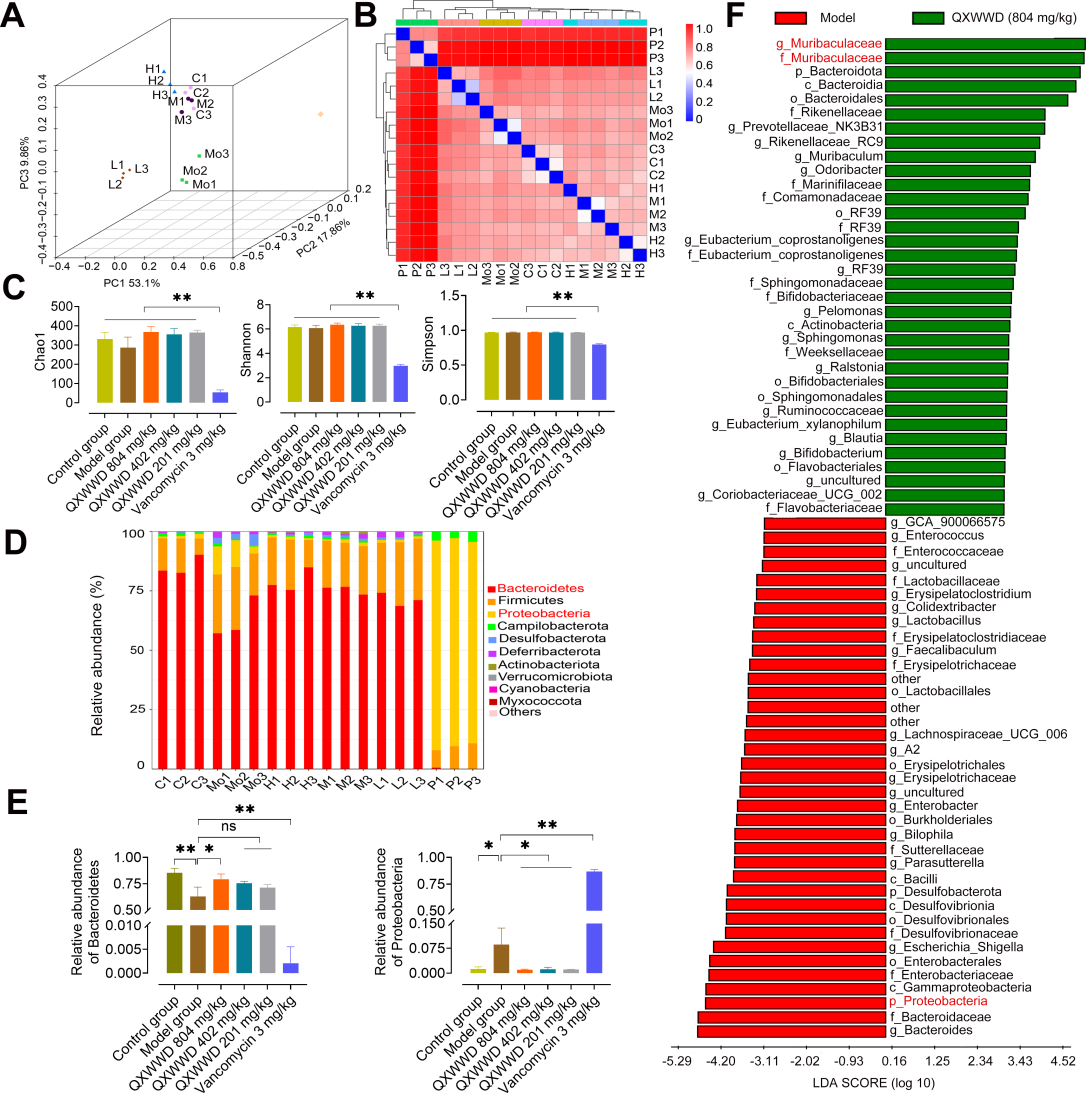
QXWWD regulates the gut microbiota composition in MRSA pneumonia mice. (A) The PCoA of the gut microbiota composition at the operational taxonomy unit level. (B) The β-diversity analysis with Bray Curtis feature event from different mouse groups. (C) Chao 1, Simpson, and Shannon indexes from different mouse groups. (D) Taxonomic distributions of gut bacterial composition at the phylum level. (E) Relative abundance of *Bacteroidetes* and *Proteobacteria* at the phylum level. (F) LEfSe analysis for differential abundant taxa detected between the model and QXWWD (804 mg/kg) groups. At least three animals were examined in each group. Control group (C1, C2, C3), MRSA-induced pneumonia model group (Mo1, Mo2, Mo3), QXWWD 804 mg/kg group (H1, H2, H3), QXWWD 402 mg/kg group (M1, M2, M3), QXWWD 201 mg/kg group (L1, L2, L3), and vancomycin 3 mg/kg group (P1, P2 P3). Data are expressed as mean ± SD with one-way ANOVA followed by the LSD multiple comparisons test. ^*^ *P* < 0.05 and ^**^ *P* < 0.01.

To explore the relationship between the relieved pneumonia symptoms and gut microbiota, the 16S rRNA from mice fecal samples were sequenced. Bray-Curtis was used to produce the principal coordinates analysis (PCoA) (Fig. 5A) which revealed that the model group assembled differently than the control or QXWWD-treated (with medium dose and high dose) clusters (*P* value = 0.001), demonstrating that MRSA infection promoted dysbiosis of gut microbiota. Furthermore, the QXWWD shielded against MRSA-induced dysbiosis because the microbiota in both high- and medium-dose groups was more closely clustered for the control group than in positive and low-dose drug groups. The β-diversity analysis (Fig. 5B) with the Bray-Curtis feature event evaluates the species' total variety within and between habitats. According to the depiction in Fig. 5B, samples that appear in blue indicate proximity and significant similarity, whereas those that appear in red signify a greater distance and lower degree of similarity between the samples. The α-diversity analysis (Fig. 5C) with Chao 1, Simpson, and Shannon indexes showed that QXWWD did not affect the bacterial diversity of the animals. The gut microbiota composition was evaluated by phylum level (Fig. 5D). MRSA infection increased *Proteobacteria*, a gut microbiota dysbiosis signature, as shown in Fig. 5E (18). The MRSA-induced reduction in the relative abundance of *Bacteroidetes* ratio was considerably boosted by QXWWD (804 mg/kg), as shown in Fig. 5E. Furthermore, the authors employed LEfSe (linear discriminant analysis effect size) to construct a histogram based on the linear discriminant analysis score. As depicted in Fig. 5F, QXWWD treatment was associated with the enrichment of some short-chain fatty acid-producing strains, such as the beneficial bacteria *Muribaculaceae* genus (19). The administration of QXWWD led to a reduction in the abundance of harmful bacteria *Proteobacteria* and an increase in the abundance of beneficial bacteria *Muribaculaceae*. These data imply that QXWWD shields against gut microbiota dysbiosis and MRSA-induced pneumonia. Furthermore, these benefits may rely on short-chain fatty acid producers.

### QXWWD’s role in gut microbiota-derived SCFAs metabolites

**Fig 6:**
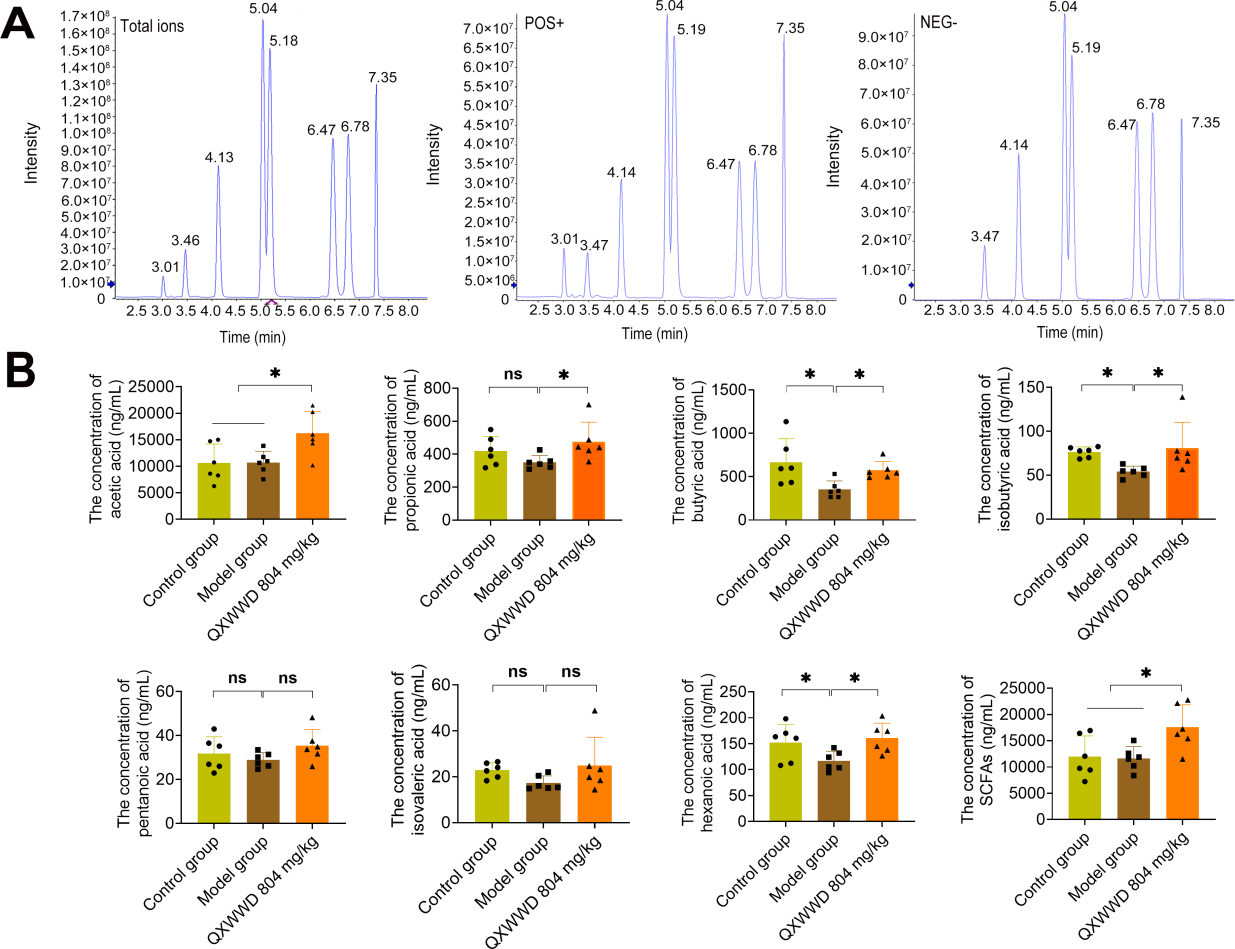
Changes in SCFA levels in the serum samples of mice with QXWWD treatment. (A) The total ion current map of the sample and the extracted ion current map of each metabolite. The abscissa is the retention time of metabolite detection, and the ordinate is the ion intensity (count per second, cps) of ion detection. (B) Concentrations of acetic acid, propionic acid, butyric acid, isobutyric acid, pentanoic acid, isovaleric acid, hexanoic acid, and total SCFAs from different groups with or without QXWWD (804 mg/kg) treatment. At least six animal samples were examined in each group. Data are expressed as mean ± SD with one-way ANOVA followed by the LSD multiple comparisons test. ^*^ *P* < 0.05.

To confirm whether the altered gut microbiota composition increased the levels of SCFAs, targeted SCFAs metabolomic data profiles were created on mice serum from control, MRSA-induced pneumonia model, and QXWWD (804 mg/kg) treatment groups by high-resolution liquid chromatography and mass spectrometry (Fig. 6A). The QXWWD (804 mg/kg) group (Fig. 6B) had higher levels of acetic, propionic, butyric, isobutyric, pentanoic, isovaleric, and hexanoic acids along with total SCFAs than the model group. Interestingly, QXWWD significantly increased acetic, propionic, butyric, isobutyric, and hexanoic acid levels and total SCFAs compared to the model groups (*P* ＜ 0.05).

### Gut microbiota from QXWWD and SCFAs-treated mice alleviates MRSA-induced pneumonia

**Fig 7:**
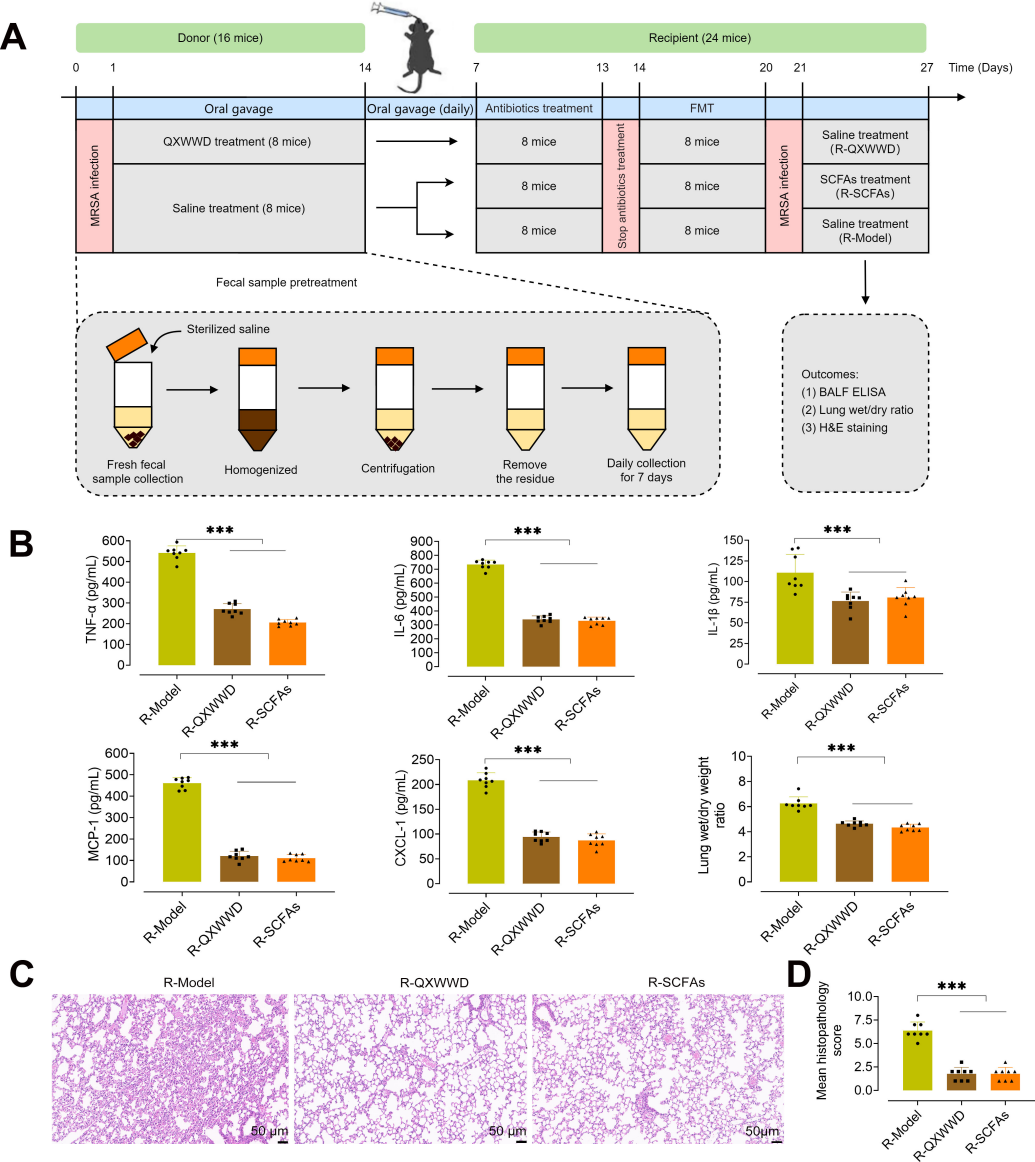
Gut microbiota from QXWWD and SCFAs-treated mice alleviates MRSA-induced pneumonia. (A) Experimental diagram of FMT and SCFAs treatment. (B) BALF TNF-α, IL-6, IL-1β, CXCl-1, MCP-1 levels, and lung *W*/*D* ratio in the feces recipient mice. (C) H&E staining and (D) histological scores of lung sections in different feces recipient mice, scale bar = 50 µm. At least eight animal samples were examined in each group. Data are expressed as mean ± SD with one-way ANOVA followed by the LSD multiple comparisons test. ^***^ *P* < 0.001.

We conducted a fecal microbiota transplantation (FMT) experiment to investigate the interaction between the gut microbiota-SCFAs axis and its effects on MRSA-induced pneumonia. A diagram of FMT and antibiotics treatment is shown in Fig. 7A. MRSA-induced pneumonia feces recipients exhibited a trend toward higher bronchoalveolar lavage fluid (BALF) TNF-α, IL-6, IL-1β, CXCl-1, MCP-1 levels, and *W*/*D* ratio (Fig. 7B). Moreover, QXWWD feces recipients and SCFAs treatment groups exhibited a trend toward lower BALF TNF-α, IL-6, IL-1β, CXCl-1, MCP-1 levels, and *W*/*D* ratio (Fig. 7B). Furthermore, there were obvious differences in lung pathology injury in FMT mice by histological analysis (Fig. 7C). MRSA-induced pneumonia recipients had higher histological scores than QXWWD feces recipients and SCFAs treatment groups (Fig. 7D). These results suggest that the gut microbiota from QXWWD-treated mice and SCFAs treatment may alleviate MRSA-induced pneumonia.

### QXWWD regulates metabolic remodeling in MRSA-induced pneumonia in mice

**Fig 8:**
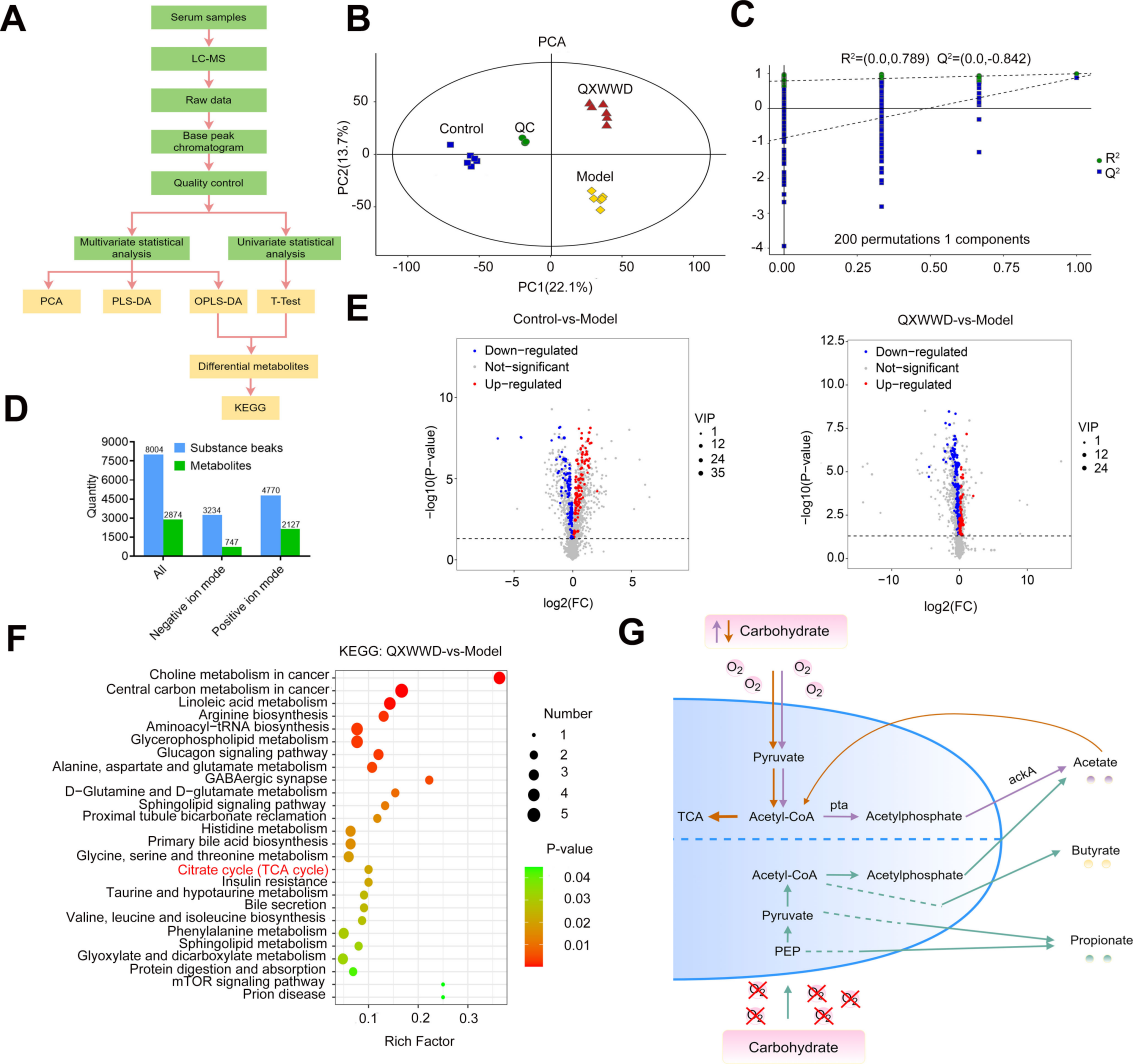
QXWWD alters metabolic remodeling and enriches the pathway of the TCA cycle. (A) Flow diagram for differential metabolites that QXWWD regulates. (B) PCA analysis of metabolites in control, MRSA-induced pneumonia model, and QXWWD (804 mg/kg) groups. (C) External verification of the OPLS-DA model. (D) A total of 2,874 metabolites were identified in mouse serum samples in both positive and negative ion modes (VIP > 1 and *P* < 0.05) with (E) 193 metabolites (control vs model), 180 metabolites (QXWWD vs model) and were significantly altered, respectively. (F) The pathway enrichment study of MRSA- and QXWWD (804 mg/kg)-treated mice serum metabolites. (G) Bacteria metabolize carbohydrates anaerobically into butyrate, propionate, and acetate. Acetate and butyrate are primarily produced via acetyl-CoA, whereas propionate is made from pyruvate or phosphoenolpyruvate (PEP) via multiple pathways. Under aerobic conditions and excess carbohydrates (orange arrows), carbohydrates are digested into acetate via acetyl-CoA using the phosphatase/acetyl-kinase A (Pta/AckA) pathway. Immune cells can recognize propionate, butyrate, and acetate produced by bacteria. Each group had six animals selected as samples for examination. The differential metabolites were measured by the combination of the PLS-DA model (VIP > 1) and the two-tailed Student’s *t*-test (*P* < 0.05) on the normalized peak intensities.

We used untargeted metabolomic analysis to assess metabolic alterations in response to QXWWD treatment to determine the variations in serum metabolites from control, MRSA-induced pneumonia model, and QXWWD (804 mg/kg) treatment groups. We expected to find the differential and related metabolic pathways through the *t*-test and orthogonal partial least-squares-discrimination analysis (OPLS-DA) that could be regulated by QXWWD (Fig. 8A). As illustrated in Fig. 8B, the principal component analysis (PCA) revealed significant segregation of data clusters among the three major mouse groups. Similarly, response permutation testing (RPT) verified that the OPLS-DA model displayed a significant performance in terms of prediction (Fig. 8C). In this study, after statistical analysis, a total of 2,874 metabolites (Fig. 8D; Table S4-1) were identified in mice serum samples in both positive and negative ion modes; 193 metabolites (control vs model, Table S4-2) and 180 metabolites (QXWWD vs model, Table S4-3) were significantly [*P* < 0.05 and variable importance in projection (VIP) > 1] altered, respectively (Fig. 8E). The metabolomic analysis map highlighted significantly enriched pathways affected by QXWWD treatment, revealing an association with several nutrient and energy metabolism pathways such as linoleic acid metabolism, TCA cycle, and amino acid metabolism (Fig. 8F). Specifically, it was found that the TCA cycle, involved in energy and SCFAs (i.e., acetic, propionic, and butyric acid) metabolism (Fig. 8G), was significantly enriched in response to QXWWD treatment.

### QXWWD alters gene expression profile and activates macrophage regulatory network

**Fig 9:**
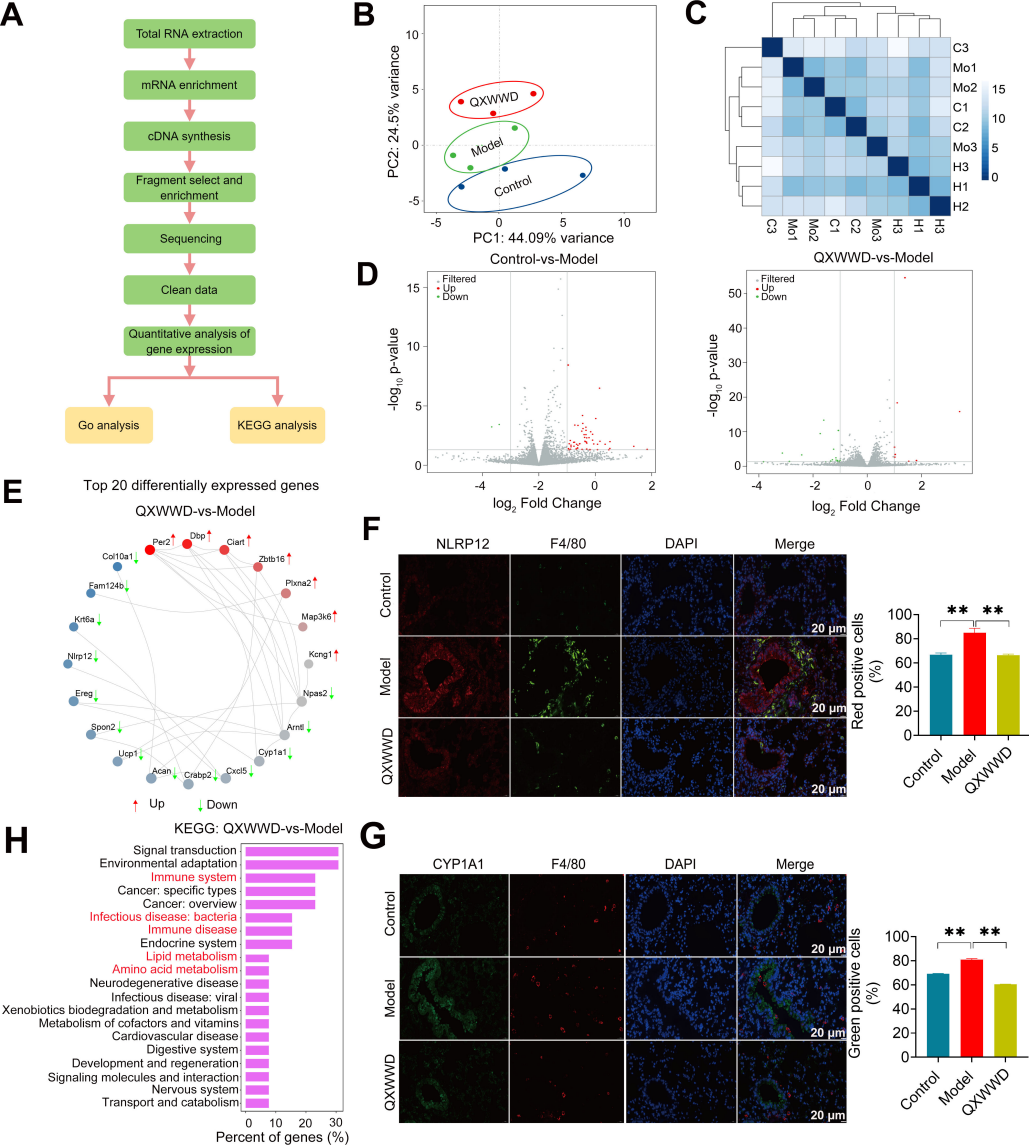
Transcriptomic analysis suggested that QXWWD regulates macrophage gene expression in multiple infections-, inflammation-, and immune-related pathways. (A) Flowchart of the identification of differential genes and pathways regulated by QXWWD. (B) PCA analysis of the differentially expressed genes in each group of mice. (C) Sample-to-sample cluster analysis results with the horizontal axis representing the sample name, the vertical axis representing the corresponding sample name, and the color representing the size of the correlation coefficient. (D) Differential gene volcano map of control vs model and QXWWD vs model mice. (E) The top 20 differentially expressed genes network of the MRSA-induced model and the QXWWD 804 mg/kg-treated mice. (F) Immunofluorescent analysis of *NLRP12* (red) and F4/80 (green) in mouse lung sections. Nuclei were stained with DAPI (blue). Scale bar = 20 µm. (G) Immunofluorescent analysis of *CYP1A1* (green) and F4/80 (red) in mouse lung sections. Nuclei were stained with DAPI (blue). Scale bar = 20 µm. (H) Differentially expressed genes regulated by QXWWD in KEGG analysis relative to the MRSA-induced model mice. At least three animal samples were examined in each group. Control group (C1, C2, C3), MRSA-induced pneumonia model group (Mo1, Mo2, Mo3), and QXWWD 804 mg/kg group (H1, H2, H3). A two-tailed Student’s *t*-test was further used to verify group differences.

To further explain the mechanisms essential for the improvements of MRSA pneumonia in QXWWD treatment mice, transcriptional profiling (genome-wide) of the primary alveolar macrophages for RNA sequencing was performed (Fig. 9A). The PCA results exhibited the expression levels of the gene in control, MRSA-induced pneumonia model and QXWWD (804 mg/kg) treatment groups showed a statistically significant distribution distance (Fig. 9B). Contrarily, the gene expression levels within samples from the same group were similar (Fig. 9C). After statistical analysis, 56 differentially expressed genes (DEGs) (control vs model, Table S5-1) and 25 DEGs (QXWWD vs model, Table S5-2) were significantly altered, respectively (Fig. 9D). The network of top 20 differentially expressed genes network also revealed that the expression level of genes between the MRSA-induced pneumonia model and QXWWD (804 mg/kg) group was significantly changed (Fig. 9E), including seven up-regulated genes (*Per2*, *Dbp*, *Ciart*, *Zbtb16*, *Plxna2*, *Map3k6*, and *Kcng1*) and 13 down-regulated genes (*Npas2*, *Arntl*, *CYP1a1*, *Cxcl5*, *Crabp2*, *Acan*, *Ucp1*, *Spon2*, *Ereg*, *NLRP12*, *Krt6a*, *Fam124b*, and *Col10a1*). Furthermore, the expression levels of two genes related to macrophage function, *NLRP12*, and *CYP1A1*, were determined by immunofluorescent staining of lung tissue. Specifically, we observed a substantial upregulation of *NLRP12* and *CYP1A1* gene expression in the model group, whereas their expression was significantly downregulated in both the control group and the group treated with QXWWD (Fig. 9F and G). The KEGG (Fig. 9H) pathway analyses of DEGs displayed the series enrichment of signaling pathways, which were closely related to immunity, infection, and nutrient and energy metabolism.

## DISCUSSION

**Fig 10:**
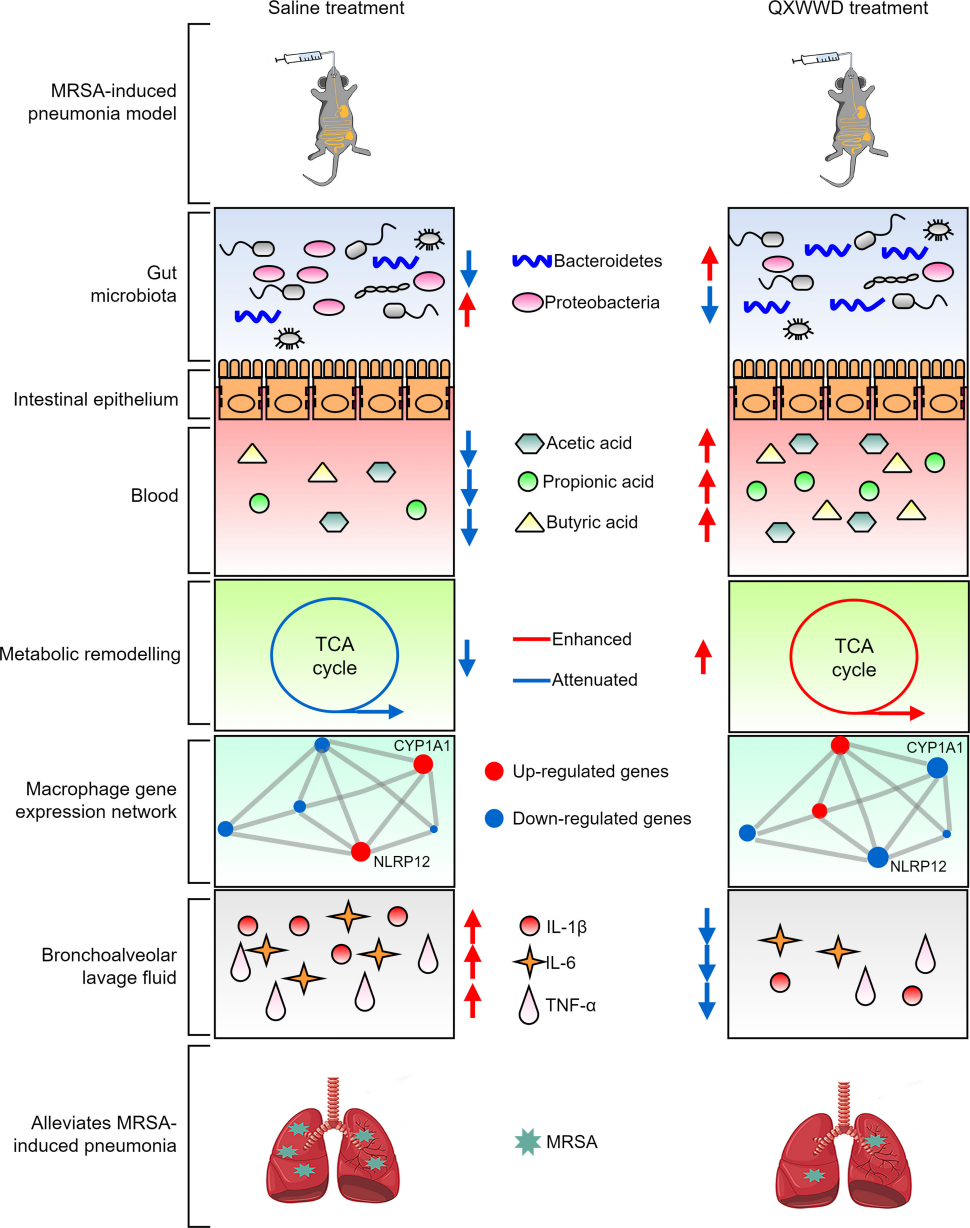
Proposed model for the anti-inflammatory and antibacterial effects of QXWWD on MRSA-induced pneumonia in mice. Treatment with QXWWD produces many beneficial changes in MRSA-induced pneumonia in mice, including increasing *Bacteroidetes* levels, the acetic, propionic, and butyric acid levels in serum, while regulating metabolic remodeling and macrophage gene expression network, reducing pro-inflammatory cytokines levels (TNF-a, IL-6, and IL-1β) in BALF, decreasing pulmonary bacterial burdens, inflammation infiltration, and the development of MRSA-induced pneumonia.

MRSA colonizes the upper respiratory airways and is antibiotic-resistant (8). MRSA is a frequently acquired infection in hospital and community settings, and it can cause conditions such as MRSA pneumonia (10). Given the rise of multidrug-resistant *Staphylococcus aureus* and the dearth of effective antibiotics, there is a need for alternative approaches to prevent or treat such infections. QXWWD shields against gut microbiota dysbiosis in mice with MRSA-induced pneumonia (9). Such changes in the composition of the gut microbiota, in turn, increase the level of acetic, propionic, and butyric acids in serum, regulate metabolic remodeling and macrophage gene expression network, reduce pro-inflammatory cytokines levels (TNF-α, IL-6, and IL-1β) in BALF, decrease pulmonary bacterial burdens, inflammation infiltration, and the development of MRSA-induced pneumonia (Fig. 10).

The gut microbiota, representing a highly complex microenvironment, plays a pivotal role in maintaining the intestinal microecological balance and regulating the immune system as part of the broader homeostasis of the human body. Recent epidemiological research (1) supports the "lung-gut axis," which coincides with the theory of TCM, i.e., "the lung-large intestine exterior-interior link." Many respiratory infections also show gastrointestinal symptoms and gut microbiota dysbiosis increases respiratory illness risk (20). TCM medicines impact gut microbiota composition. The gut microbiota metabolizes carbohydrates, proteins, lipids, and non-nutritive small chemical components from TCM herbs into chemical metabolites (21). The gut microbiota composition modulation may contribute to the disease-relieving effects of TCMs. The *Firmicutes*, *Bacteroidetes*, *Verrucomicrobia*, *Proteobacteria*, *Deferribacteres*, *Actinobacteria*, and *Cyanobacteria* were most impacted (22). Ingestion of various TCM herbs can potentially alter the balance between beneficial (anti-inflammatory, SCFA-producing) and detrimental (pro-inflammatory, pathogenic) bacteria (23). Altering gut microbiota composition is linked to host immunological and metabolic activity.

The gene sequencing of 16S rRNA showed that mice treated with MRSA had a much lower ratio of *Bacteroidetes*, which the QXWWD effectively rescued. The dysbiosis-associated reduction in gut microbial diversity may be due to a shift in the balance between commensal and potentially pathogenic microorganisms. Previous studies revealed that anaerobic *Bacteroidetes* and *Firmicutes* dominate a healthy gut microbiota, while the expansion of *Proteobacteria* introduces microbiota dysbiosis (22). The results also revealed that the QXWWD dramatically decreased *Proteobacteria* in MRSA-treated mice. QXWWD treatment was associated with the enrichment of some short-chain fatty acid-producing strains, such as the beneficial bacteria *Muribaculaceae* genus. It was also observed that the serum SCFAs, specifically acetic, propionic, and butyric acid levels, were also elevated. Gut microbiota from QXWWD and SCFAs-treated mice alleviates MRSA-induced pneumonia. Butyrate may be a local energy source for colon cells (5). Activating anti-inflammatory Treg cells reduces low-grade inflammation (24) and decreases pro-inflammatory chemokines and cytokines (25). GPR43 is a G protein-coupled receptor that resides predominantly on the cell membranes of specific cell types, most notably within the gastrointestinal tract and immune cells (5). Its activation hinges on the interaction with SCFAs (5). Acetate and propionate are the most effective activators of GPR43 (5). The neutrophils, leukocytes, and enterocytes express the GPR43 (5). These results are also consistent with recent findings showing that targeted activation of GPR43 reduces susceptibility to infection by bacteria such as *Staphylococcus aureus*, *Citrobacter rodentium*, and *Klebsiella pneumonia*, as well as viruses like respiratory syncytial and influenza viruses (26). Thus, our immune system may detect SCFAs dependent on GPR43 to avoid and identify severe infections (27). Recent progress in understanding the function of SCFAs and GPR43 in diverse infections and suggesting manipulating them as a therapeutic method to combat infections were highlighted.

The untargeted mice serum metabolomics analysis from various treatment groups was performed to evaluate the impact of changed gut microbiota on host metabolism. It indicated that microbiota-SCFAs regulated metabolic remodeling by promoting the TCA cycle. Due to immune cells' O_2_ consumption and nutrition during immunological responses, abscesses are generally hypoxic and have limited glucose (28). This scenario inhibits bacterial energy synthesis via the TCA cycle and glycolysis, leading to the utilization of amino acids (29) and the production of SCFAs. The abscesses and infection sites have elevated SCFA concentrations, notably acetate (30). This is also true for mastitis (31), endometritis (32), chronic rhinosinusitis (33), and atopic dermatitis (34) due to *Staphylococcus aureus* infection.

Macrophages manage infections by phagocytosing, presenting antigens, and producing cytokines (35). So far, two macrophage subpopulations have been recognized, i.e., the inflammatory or classically activated M1 and M2 (anti-inflammatory) macrophages (36). M1 macrophages are triggered by trauma or infections and move to wounded regions to eradicate the stimulus signal by cytokine production and cytotoxic activities. Over-activation of macrophages can potentially lead to a cytokine storm and excessive inflammation. Through the application of network pharmacology supported by mass spectrometry, it was discerned that QXWWD exhibits regulatory impacts on inflammation, infection, and immunity. Furthermore, investigating QXWWD’s influence in a pneumonia mouse model confirmed its anti-inflammatory and antimicrobial attributes. A wide array of gene alterations was identified during transcriptome analysis, including the genes nucleotide-binding oligomerization domain-like receptors protein-12 (*NLRP12*) and cytochrome P450 1A1 (*CYP1A1*), involved in the immune response mediated by macrophages. A previous study revealed that *NLRP12* is a significant bacterial promoter (*Lachnospiraceae*), had a negative regulator inflammatory response, and sustained intestinal SCFA-producing bacteria that significantly prevented the associated diseases and excessive inflammation (36). In contrast to pro-inflammatory immune receptors, the proteins of NLR diminished inflammation, which is very important for the resolution of inflammation. Among them, *NLRP12* is identified as a potent mitigator of inflammation. It is primarily expressed by dendritic cells, granulocytes, and macrophages, inhibiting canonical and noncanonical nuclear factor kappa-B (NF-kB) and extracellular signal-regulated kinase (ERK) activation (37 – 39). *CYP1A1* gene within macrophage is associated with its antibacterial and immune activity. Specifically, we observed a substantial upregulation of *CYP1A1* gene expression in the model group, whereas its expression was significantly downregulated in both the control group and the group treated with QXWWD. These findings hold significant implications and provide intriguing insights into the potential involvement of the *CYP1A1* gene in governing the phagocytic and bactericidal functions of macrophages. Furthermore, our preliminary cell phenotype experiments yielded compelling results that suggest a potential linkage between the *CYP1A1* gene and the aforementioned functions of macrophages. These findings open new avenues for exploring the underlying molecular mechanisms through which *CYP1A1* influences macrophage activity.

A typical TCM is composed of multiple components and has the characteristics of multiple targets. Notably, top components (eupatilin, apigenin, luteolin, quercetin, and rhapontigenin) of network pharmacology sorted by degree value within QXWWD have demonstrated a notable propensity to wield their healing influences across a spectrum of ailments, extending even to the intricate realm of cancer, thus accumulating in some cancer-related pathways (such as Kaposi sarcoma-associated herpesvirus infection and pathways in cancer). As a promising TCM, in the future, we will pay attention to its therapeutic effect on other diseases, such as cancer.

This research shows that TCM reprograms and corrects the serum metabolism and macrophage gene expression in the distal lung through the microbiota-SCFAs axis. A typical TCM formula like QXWWD has many compounds, each of which may create hundreds of metabolites. Whether additional gut microbiota-derived components and metabolites contribute to these effects is unknown. In addition, the gut microbial SCFAs and the host internal effector gene (e.g., *NLRP12* and *CYP1A1*) control the host immune system and require further study. It remains unclear whether the impact of gut microbiota on MRSA infection extends to other pneumonia-causing pathogens. Furthermore, characterizing microbial indicators can be an effective strategy for translating microbiome research into therapeutic practice. Based on these observations, exploring future novel medicines and diagnostic biomarkers targeting the gut microbiota may be useful for refractory bacterial pneumonia therapy.

### Conclusions

Using a combination of multi-omics techniques, such as mass spectrometry-guided network pharmacology, 16S rRNA sequencing, targeted serum SCFAs, FMT, untargeted metabolomics, and macrophage transcriptome sequencing, the present investigation unveiled the roles of QXWWD in the management of MRSA-induced pneumonia in murine models via the microbiota-SCFAs pathway. These findings also shed light on novel perspectives about the fundamental mechanisms and components of QXWWD involved in the pathological processes of MRSA-induced pneumonia. Additionally, this study helps illuminate the prospective therapeutic potential that QXWWD presents for managing resistant bacterial pneumonia.

## MATERIALS AND METHODS

### Materials and reagents

Individual herbs of QXWWD were all obtained from the market of Anguo (China). The acetic, propionic, butyric, isobutyric, pentanoic, isovaleric, and hexanoic acids standards were purchased from Sigma-Aldrich (USA). Similarly, formic acid, methanol, and HPLC (high-performance liquid chromatography)-grade-acetonitrile were provided by Fisher (USA). The MRSA (ATCC43300) and MH-S cells were procured from ATCC (USA). RPMI1640 medium, fetal bovine serum (FBS), and antibiotics were obtained from Gibco (USA). Enzyme-linked immunosorbent assay (ELISA) kits were obtained from Sigma-Aldrich (USA).

### QXWWD preparation

*Sophorae flavescentis radix* (Ks, 18 g), *Fructus cnidii* (Scz, 18 g), *Rhei Radix et Rhizoma* (Dh, 18 g), and *Artemisia Argyi Folium* (Ay, 18 g) were dipped in 70% ethanol (720 mL) for 30 min. Afterward, they were refluxed twice for 1 h and filtered. The filtrate was concentrated and collected to achieve a final 1 g raw herb/mL concentration. After then, *Borneolum* (Bp, 0.9 g) was added to the filtrate for the high-resolution mass spectrometry analysis. All extracts were concentrated and conserved for animal trials by freeze-drying and lowering the pressure.

### High-resolution mass spectrometry analysis of QXWWD

The QXWWD extract was centrifuged for 15 min at 1 × 10^4^ rev/min. The supernatant was collected and subsequently diluted with methanol (50%). The solution was filtered with a filter (0.22 µm) for the high-resolution mass spectrometry analysis. The Thermo U3000 system (Thermo Fisher, USA) that was equipped with an electrospray ionization (ESI) source was used for chemical identification. The samples were separated on a Shim-pack GIST-HP C18 (4.6 mm × 150 mm, 3 µm) at a flow rate of 1 mL/min, with the column temperature set at 40°C and an injection volume of 20 µL. Furthermore, the mobile phases were as follows: formic acid-water (0.1%) was used as an aqueous mobile phase (A), and formic acid-acetonitrile (0.1%) was used as an organic mobile phase (B). Similarly, the gradient procedure was 0–10 min, 10%–10% A; 10–15 min, 10%–45% A; 15–20 min, 45%–60% A; 20–25 min, 60%–75% A; 25–30 min, 75%–90% A; 30–40 min, 90%–90% A. The MS analysis was performed in both modes (negative and positive) for the mass-to-charge ratio (*m*/*z*) scan ranges 110–1,200, positive ion detection mode (auxiliary gas volume flow rate 30 L/min, spray voltage setting 3.50 kV, ion transfer tube temperature 300°C, auxiliary gas temperature 200°C, and collision energy 45 eV); negative ion detection mode (auxiliary gas volume flow rate 30 L/min, spray voltage 2.80 kV, ion transfer tube temperature 400°C, auxiliary gas temperature 100°C, and collision energy 30 eV).

### High-resolution mass spectrometry-based compound-target network of QXWWD

The targets associated with the QXWWD compound in mass spectrometry were sourced from the SEA database (https://sea.bkslab.org/) and Swiss Target Prediction (http://www.swisstargetprediction.ch/). These targets have been designated as QXWWD-associated components. For the selection of linked targets, the search term “bacterial pneumonia” was employed while exploring the Disgenet (https://www.disgenet.org/), GeneCards (https://www.genecards.org/), and OMIM databases (https://omim.org/), leading to the identification of targets termed as “bacterial pneumonia”-related targets. Through the amalgamation of these two categories, a total of 126 shared genes were derived. These common genes, originating from both the QXWWD-associated component targets and the “bacterial pneumonia”-related targets, along with the associated compounds, were visualized using Cytoscape (version 3.8.0) to construct a comprehensive compound-target network. Moreover, the overlapping genes shared between QXWWD component-related targets and “bacterial pneumonia”-related targets were integrated into the STRING database (https://string-db.org/cgi/input.pl) to construct a PPI network. The Cytoscape (version 3.8.0) was used to construct and visualize the PPI network. Furthermore, functional enrichment, including GO (Gene Ontology) and KEGG of the analysis of the critical target, was performed by the David database (https://david.ncifcrf.gov/).

### MRSA-induced mouse pneumonia model and QXWWD treatment

In the present study, proper guidelines were followed as permitted by the Animal Protection and Use Committee of Inner Mongolia Medical University (approval number: YKD202201017). The experiment involved female mice (C57BL/6), aged between 6 and 8 weeks and weighing from 15.91 to 18.71 g, provided by SPF Biotechnology Co., Ltd (China). These mice were randomly assigned to one of the six groups: control, model (MRSA-induced pneumonia), high dose of QXWWD, medium dose of QXWWD, low dose of QXWWD, and positive drug (vancomycin), each comprising 20–25 mice. The MRSA**-**induced pneumonia mouse model was designed as previously described (40, 41). Isoflurane (1.5%) was used for anesthesia, and mice were inoculated with 1 × 10^9^ CFU/30 µL/mouse. The MRSA was injected via the nasal route for lung tissue infection. At 24 h after inoculation, sterile saline, QXWWD (high dose: 804 mg/kg, medium dose: 402 mg/kg, and low dose: 201 mg/kg), and vancomycin 3 mg/kg were orally given to the mice daily for 14 days. The dosage of QXWWD (low dose) used in mice was converted from clinical dosage as previously stated (42). Furthermore, the body weight was also determined to evaluate the mice’s general characteristics during experiments.

### Lung wet-to-dry weight ratio

The right lung was removed and rinsed in sterile phosphate-buffered saline (PBS) to obtain the wet weight (*W*). The right lung was oven-dried at 60°C to get the dry weight (*D*). The wet-to-dry weight ratio (*W*/*D*) was calculated as *W*/*D*.

### BALF collection and ELISA

After the mice were anesthetized and euthanized, the thorax and trachea were exposed, the main trachea and the right pulmonary bronchus were ligated, and the left lung of the mice was irrigated with 0.35 mL ice-cold PBS three times. The BALF was centrifuged at 800 rev/min for 5 min at 4°C. The supernatant was collected and stored in a −80°C freezer until further use. The IL-6, TNF-α, MCP-1, CXCl-1, and IL-1β concentrations in BALF were observed by ELISA kits according to the manufacturer’s instructions.

### CFU and hematoxylin-eosin staining

Under the aseptic condition, the tissue samples were taken after the mice were sacrificed and resected. The left lung tissues were homogenized, and the CFU was measured by the successive dilution approach on agar plates. Similarly, the right lung tissues were fixed in the formalin (10%), followed by sectioning and finally staining with hematoxylin-eosin (H&E).

### Immunohistochemistry and immunofluorescence

Following deparaffinization and rehydration, a 3% bovine serum albumin (BSA) (Servicebio, China) was used to block the lung sections at room temperature for half an hour, and then PBS was used for washing the sample. For immunohistochemistry analysis, the primary antibody F4/80 (#GB113373, Servicebio, China) and the tissue sections sample were incubated at 4°C overnight. All the slides were cleaned by washing in PBS solution three times before applying peroxidase-conjugated secondary antibody (#G1213, Servicebio, China) at room temperature for about 2 h. For immunofluorescence staining, the paraffin-embedded sections of the lung were incubated with primary antibodies of F4/80 (#GB113373, Servicebio, China), *NLRP12* (#A6671, ABclonal, China), *CYP1A1* (#13241-1-AP, Proteintech, China), *iNOS* (#GB11119, Servicebio, China), and *CD20*6 (#GB113497, Servicebio, China) overnight at 4°C. The next day, horseradish peroxidase marked goat anti-rabbit IgG secondary antibodies (#GB25303; #GB21303, Servicebio, China) were used. The slides were placed in PBS (pH 7.4), washed three times on a decolorization shaker, and stained with a TSA kit (#G1235, Servicebio, China) for 10 min. Nuclei were incubated with DAPI (#G1012, Servicebio, China) solution at room temperature for 10 min.

### Survival analysis

For the survival analysis, eight mice in every group were anesthetized and infected with 2 × 10^9^ CFU/30 µL/mouse MRSA. At 24 h after inoculation, QXWWD (804 mg/kg, 402 mg/kg, and 201 mg/kg) and vancomycin 3 mg/kg were orally given to the mice every 24 h to evaluate the 96-h survival rates.

### 16S rRNA gene sequencing

Mice fecal samples were snap-frozen and were stored at a cold temperature (−80°C) after sterile collection. The DNA (genomic) was then extracted using a commercially available kit, i.e., MagPure Soil DNA-LQ kit (Magen, China) with the user’s protocol. Furthermore, the NanoDrop (model: 2000, Thermo Fisher, USA) was used to check DNA concentrations. Similarly, the quantification was also done on the agarose gel. For bacterial diversity analysis, amplification of the variable regions of the 16S rRNA V3-V4 genes was conducted using forward primer 343 F: 5′-TACGGRAGGCAGCAG-3′ and reverse primer 798 R: 5′-AGGGTATCTAATCCT-3′. Furthermore, the Qubit dsDNA assay kit (Life Technologies, USA) was used for amplicon quantification. The purified and an amount of amplicon were grouped for subsequent sequencing using the MiSeq platform (Illumina).

The raw sequencing data, in FASTQ format, underwent bioinformatics analysis. Using the software tool cutadapt, paired-end reads were initially processed to identify and excise the adapter. Following this process, paired-end reads underwent low-quality sequence filtering, denoising, merging, and chimera read removal with DADA2 (43) aid, adhering to QIIME2’s standard parameters (44). The software consequently generated representative reads and an ASV abundance table. The QIIME2 package was used to analyze the representative reads of each ASV. Moreover, all reads were annotated and subjected to a blast procedure using Q^2^-feature-classifier, in line with the default parameters, against the Silva database (version 138).

### Measurement of serum SCFAs

The serum sample collection was done after the mice were anesthetized and sacrificed. Subsequently, the collected samples were stored at a cold temperature (−80°C) and were thawed at room temperature before use. A total of 100 µL of the sample was poured into an Eppendorf tube (1.5 mL) with (2H^9^)-pentanoic acid and (2H^11^)-hexanoic acid dissolved in 80 µL 50% acetonitrile-water solution as internal standard. After that, the tubes were thoroughly vortexed for about 10–15 s. The samples were extracted by ultrasonic method for 10 min in an ice-cold-water bath. After that, they were stored at −20°C for half an hour. The sample was then centrifuged (with a temperature of 4°C) at 13,000 rev/min for 10 min. Subsequently, a 40 µL from each of the 200 mM 3-NPH (3-nitrophenylhydrazine) and 120 mM EDC [1-ethyl-3-(3-dimethyl aminopropyl) carbodiimide]-6% pyridine were added to 80 ul of supernatant and were placed for 30 min at an ambient temperature. The supernatants were collected with crystal syringes, filtered through microfilters (0.22 µm), and shifted to LC vials until LC-MS analysis. Furthermore, the preparation of quality control (QC) samples was done by mixing all the sample’s aliquots to make a pooled sample.

The HPLC analysis was performed using the ACQUITY UPLC BEH C18 column (100 × 2.1 mm, 1.7 µm; Waters, USA), with the column temperature set at 40°C and an injection volume of 5 µL. The linear gradient elution flow rate was 0.35 mL/min. The formic acid (0.1%) and water solution were used as mobile phase A, while the acetonitrile was used as mobile phase B. The elution gradient procedure was as follows: 0 min A/B, 1 min A/B (90:10, vol/vol), 2 min A/B (75:25, vol/vol), 6 min A/B (65:35, vol/vol), 6.5 min A/B (5:95, vol/vol), 7.8 min A/B (5:95, vol/vol), 7.81 min A/B (90:10, vol/vol), 8.5 min A/B (90:10, vol/vol). Subsequently, MS analysis was conducted in both positive and negative modes, with parameters such as spray gas (psi: 50), auxiliary heating gas (psi: 60), curtain gas (psi: 35), collision-activated dissociation parameters set at medium, and positive and negative ion spray voltages of 5,500 V and −4,500 V, respectively. The ion source temperature was set at 450°C.

UPLC-ESI-MS/MS (Shimadzu LC-30A, Japan) was used to quantitatively analyze acid (propionic, acetic, butyric, pentanoic, isobutyric, hexanoic, and isovaleric) along with total SCFAs according to the peak area of the metabolite and related standard curve. All mixed standard solutions were progressively diluted by a factor of 2 in 75% aqueous methanol, resulting in final concentrations ranging from 20 g/mL to 0.005 g/mL for each metabolite. The propionic, acetic, butyric, pentanoic, isobutyric, hexanoic, and isovaleric acids for accurate quantitation details of the analysis had been previously published elsewhere (45).

### FMT and SCFAs treatment

The modified method described previously was used to perform FMT (46). Briefly, we collected fecal samples on day 14 post-QXWWD administration. The gut microbiota was eliminated by antibiotic treatment by oral gavage in recipient mice for 7 days. Next, feces pellets were suspended and homogenized in sterile PBS at 0.125 g/mL before centrifugation (46). The resulting fecal supernatant was administered to the recipient mice daily via oral gavage for 7 days, after which they were subjected to MRSA infection. At 24 h after inoculation, mice were pretreated with sterile saline or SCFA mixtures by oral gavage for 7 days. Furthermore, the lung *W*/*D* ratio, BALF collection, and ELISA and H&E staining were also determined to evaluate the therapeutic effects of the microbiota-SCFAs axis on MRSA-induced pneumonia.

### Non-targeted metabolomics

The stored (−80°C) serum samples were first thawed at room temperature, and then the sample (100 µL) was poured into an Eppendorf tube (1.5 mL) that was previously added 20 µL of L-2-chlorophenyl alanine (0.3 mg/mL) which was dissolved in the methanol (internal standard). Subsequently, the tubes were vortexed for approximately 10 s. Afterward, 300 µL of methanol and acetonitrile (ice-cold mixture with vol/vol = 2/1) was added. The samples were ultrasonically extracted for 10 min in an ice-water bath and kept at −20°C for 30 min. The extract obtained was centrifuged for about 10 min at 4°C with 13,000 rev/min. A total of 200 µL of supernatant in a glass vial was freeze-dried, and 300 µL of methanol and water with the ratio of 1/4 (vol/vol) was mixed with all the samples, vortexed for 30 s, ultrasonically extracted for 3 min in an ice-water bath, and then frozen for 2 h. The samples were then centrifuged at 13,000 rpm for 10 min at 4°C. The 150 µL supernatants were taken from each tube, filtered using 0.22 µm microfilters, and transferred to LC vials. The vials were stored at a cold temperature (−80°C) until analyzed by LC-MS. Similarly, the QC samples were produced by mixing all the sample’s aliquots to make a pooled sample.

A Dionex Ultimate 3000 RS UHPLC (USA) fitted with Q-Exactive plus quadrupole-orbitrap mass spectrometer (USA) equipped with heated ESI source (Waltham, MA, USA) was used to perform the metabolic profile analysis in both the ions modes, i.e., ESI positive and ESI negative. Similarly, both positive and negative modes used the ACQUITY UPLC HSS T3 column (1.8 µm, 2.1 × 100 mm). The gradient elution system consisted of (A) water and formic acid (0.1% concentration) and (B) acetonitrile formic acid (0.1% concentration). The separation was executed according to the following gradient protocol: 0 min A/B, 2 min A/B (95:5, vol/vol), 4 min A/B (70:30, vol/vol), 8 min A/B (50:50, vol/vol), 10 min A/B (20:80, vol/vol), 14 min A/B (0:100, vol/vol), 15 min A/B (0:100, vol/vol), 15.1 min A/B (95:5, vol/vol), and 16 min A/B (95:5, vol/vol). During the entire course of the experimental analysis, all samples were maintained at a temperature of 4°C. Meanwhile, the column temperature was sustained at 45°C, and a flow rate of 0.35 mL/min was implemented. The volume of the injection was 2 µL. Similarly, the mass ranged from 100 to 1,000. The full 17,500 for HCD MS/MS and MS scans resolution was set at 70,000. The energy level was set at 10, 20, and 40 eV for collision. The mass spectrometer operated as follows: spray voltage of 3,800 V (positive) and 3,000 V (negative), with a gas flow rate of the sheath at 35 arbitrary units; gas flow rate of the auxiliary was at 8 arbitrary units; the temperature of the capillary was 320°C; the temperature of the Aux gas heater was 350°C; S-lens RF level 50. The QCs were injected regularly throughout the analytical run to provide data sets to assess the repeatability.

The baseline filtering, normalization, integral, retention time correction, peak identification, and alignment were performed on the data of LC-MS using the program Progenesis (QI version 2.3). The self-built databases, Metlin (Tandem and Metabolite MS Database), Lipidmaps (version 2.3), and Human Metabolome Database were used to identify compounds based on the exact mass-to-charge ratio (*m*/*z*), isotopic distributions, and secondary fragments. The matrix was imported into R, which performed PCA to observe the samples' overall distribution and the analysis process’s general stability. The metabolites that differ among the groups were identified by the partial least squares discriminant analysis (PLS-DA) and OPLS-DA. A 200-RPT and sevenfold cross-validation were used to assess the model quality to prevent overfitting. The OPLS-DA model’s VIP values was considered for grading the total impact of every parameter on the group discrimination. The *t*-test was also utilized to see whether the groups' metabolite differences were significant. The VIP values for differential metabolites were selected based on statistical analysis (VIP > 1.0 and *P* values < 0.05).

### Isolation of alveolar macrophages

As previously stated (47), alveolar macrophages were isolated. The mice were anesthetized and then sacrificed. BALF was collected with 4 mL of 37°C sterile PBS containing 0.5 mM EDTA. The cells were pelleted and reconstituted in RPMI 1640 supplemented with 10% FBS before adhering to a tissue culture flask for 2 h. Flow cytometry analysis revealed that alveolar macrophage purity was more than 90%.

### RNA sequencing

The total RNA of alveolar macrophages was extracted with the commercially available kit, miRNA Isolation Kit of mirVana (Ambion, USA), with recommended protocol. The Bio-analyzer (Agilent 2100, USA) was used to evaluate the RNA integrity. The samples with RNA integrity number ≥7 were subjected to further analysis. The mRNA LTSample Prep Kit of TruSeq Stranded provided by Illumina (USA) was used according to the manufacturer’s protocol to construct the libraries. The libraries were also sequenced (Illumina, HiSeqTM 2500), and 150 bp paired-end reads were generated.

The trimmomatic was used to clean up raw data (raw readings). The clean readings were obtained by removing ploy-N-containing, and low-quality reads. We used Hisat2 to map the clean reads to the reference genome (48). The read counts of each gene were acquired using htseq-count, and the fragments per kilobase of exon model per million mapped fragments value was determined using cufflinks (49). The R package functions (DESeq) to estimate the nbinom test and size factors were used to identify DEGs. The criterion for substantially differential expression was established at a *P* < 0.05 and |LogFC| > 1. To investigate gene expression patterns, a DEGs hierarchical cluster analysis was used. The hypergeometric distribution was used to analyze the KEGG pathway and GO enrichment DEGs using R.

### Data analysis

All data are represented as mean ± standard deviation. The two-tailed *t*-test was used to evaluate the significant differences between the two groups. Similarly, one-way analysis of variance was used to conduct multiple group comparisons (by multiple comparisons test) and least significant difference. The Mantel-Cox test was used to evaluate the difference in survival among groups of mice within 96 h. The GraphPad (version PRISM 8.0) or SPSS (version SPSS 20.0) software was used to analyze all the data. *P* < 0.05 was considered the selection criterion for statistical significance.

## Data Availability

The sequence dataset generated in this study has been deposited in the NCBI Sequence Read Archive (SRA) database (PRJNA980576, PRJNA985872). All data generated during this study are included in the article and its supplementary information files.

## SUPPLEMENTAL MATERIAL

Table S1 spectrum.00344-23-s0001.doc Compound list for QXWWD identified by HPLC-Q-Exactive-MS.

Table S2 spectrum.00344-23-s0002.xls GO enrichment.

Table S3 spectrum.00344-23-s0003.xls KEGG enrichment.

Table S4 spectrum.00344-23-s0004.xlsx Substance peaks and metabolites.

Table S5 spectrum.00344-23-s0005.xlsx Differentially expressed genes.

ASM does not own the copyrights to Supplemental Material that may be linked to, or accessed through, an article. The authors have granted ASM a non-exclusive, world-wide license to publish the Supplemental Material files. Please contact the corresponding author directly for reuse.
