# Supplementary material for: QingXiaoWuWei decoction alleviates methicillin-resistant Staphylococcus aureus-induced pneumonia in mice by regulating metabolic remodeling and macrophage gene expression network via the microbiota-short-chain fatty acids axis
Source: Microbiol Spectr. 2023 Oct 12;11(6):e00344-23. doi: 10.1128/spectrum.00344-23 (PMC10714818; doi:10.1128/spectrum.00344-23)
Supplement: Table S1 — Compound list for QXWWD identified by HPLC-Q-Exactive-MS. [file spectrum.00344-23-s0001.doc]

**Table S1 Compound list for QXWWD identified by HPLC-Q-Exactive-MS**

| **No.** | **tR/min** | **Molecular**  **Formula** | **ESI-MS** | **Error**  **(ppm)** | **ESI-MS/MS** | **Identity** | **Category** |
| --- | --- | --- | --- | --- | --- | --- | --- |
| 1 | 1.69 | C_7_H_12_O_6_ | [M-H]^-^ | 2.018 | 191.05540 | Quinic acid | Organic acids |
| 2 | 1.72 | C_5_H_11_NO_2_ | [M+H]^+^ | -2.838 | 118.08592, 72.08125 | Valine | Amino acids |
| 3 | 2.53 | C_4_H_7_NO_4_ | [M-H]^-^ | -1.168 | 132.02898 | Acidum asparticum | Amino acids |
| 4 | 3.55 | C_7_H_6_O_5_ | [M-H]^-^ | 0.475 | 169.01323, 125. 02317 | Gallic acid | Organic acids |
| 5 | 3.68 | C_15_H_24_N_2_O | [M+H]^+^ | -4.053 | 249.19513 | Matrine | Alkaloids |
| 6 | 4.34 | C_15_H_22_N_2_O | [M+H]^+^ | -3.371 | 247. 1812, 148. 11136,  179. 15448, 136. 11269, | Sophocarpine | Alkaloids |
| 7 | 5.16 | C_15_H_22_N_2_O_2_ | [M+H]^+^ | -2.753 | 136. 11214, 150. 12706,  245. 16432, 98. 09676,  203. 11726, | Oxysophocarpine | Alkaloids |
| 8 | 5.47 | C_15_H_24_N_2_O_2_ | [M+H]^+^ | -2.355 | 265.19043, 247. 18015, 148. 11134, 205. 13290 | Oxysophoridine | Alkaloids |
| 9 | 5.57 | C_15_H_24_N_2_O_2_ | [M+H]^+^ | -3.260 | 265.19019, 247. 17992,  162.22079, 205. 13284,  148. 11131, 136. 11255 | Oxymatrine | Alkaloids |
| 10 | 8.54 | C_16_H_18_O_9_ | [M-H]^-^ | 4.479 | 353.08829, 191.05536,  179.03406, 173.04462 | Chlorogenic acid | Organic acids |
| 11 | 8.57 | C_15_H_22_N_2_O | [M+H]^+^ | -3.236 | 247.17969, 148. 11160, 112. 07607, 176. 10686, | 12, 13-didehydromatridin-15-one | Alkaloids |
| 12 | 8.91 | C_12_H_16_N_2_O | [M+H]^+^ | -2.972 | 205.13293, 146. 05962, | N-Methylcytisine | Alkaloids |
| 13 | 10.53 | C_15_H_14_O_6_ | [M-H]^-^ | 4.896 | 245.08177, 231.02989,  205. 05003 | (+)-Catechin | Flavonoids |
| 14 | 11.92 | C_26_H_28_O_14_ | [M-H]^-^ | 2.057 | 563.14069, 473.10883,  443.09839, 383.07745,  353.06674 | Schaftoside | Flavonoids |
| 15 | 12.81 | C_23_H_26_O_11_ | [M-H]^-^ | 3.379 | 477.14075, 313.05670,  271. 06226 | Lindleyin | Phenols |
| 16 | 12.83 | C_27_H_30_O_16_ | [M-H]^-^ | 2.405 | 609.14661, 301.03470,  300.02762 | Rutin | Flavonoids |
| 17 | 13.17 | C_22_H_18_O_10_ | [M-H]^-^ | -4.430 | 441.08011, 169.01312 | (-)-Epicatechin gallate | Tannins |
| 18 | 13.20 | C_21_H_20_O_12_ | [M-H]^-^ | 1.960 | 463.08801, 300.02747,  287.05624, | Hyperoside | Flavonoids |
| 19 | 13.48 | C_25_H_24_O_12_ | [M-H]^-^ | 0.713 | 515.11877, 353.08826,  335.07782, 191.05539,  173.04469, 161.02341 | 1, 3-Dicaffeoylquinic acid | Organic acids |
| 20 | 13.51 | C_15_H_14_O_4_ | [M-H]^-^ | -0.916 | 257.08060, 215.12822, | Rhapontigenin | Anthraquinones |
| 21 | 13.89 | C_21_H_20_O_11_ | [M-H]^-^ | 2.600 | 447.09335, 285.03903,  284.03278, 241.05005,  151.000253, | Cynaroside | Flavonoids |
| 22 | 15.54 | C_11_H_14_N_2_O | [M+H]^+^ | -2.300 | 191.11745, 148. 07510, 133. 10089, 146. 06003,  162. 05424, | Cytisine | Alkaloids |
| 23 | 15.82 | C_22_H_18_O_11_ | [M-H]^-^ | -2.139 | 457.07556 | Epigallocatechin gallate | Tannins |
| 24 | 15.84 | C_21_H_22_O_9_ | [M-H]^-^ | 1.681 | 417.11871 | Aloin | Anthraquinones |
| 25 | 15.90 | C_15_H_10_O_4_ | [M+H]^+^ | -3.510 | 255.06429, 227. 06870,  199. 07503 | Chrysophanol | Anthraquinones |
| 26 | 16.39 | C_21_H_20_O_10_ | [M-H]^-^ | 2.660 | 431.09842, 271.05078 | Aloe-emodin-8-O-beta-D-  glucopyranoside | Anthraquinones |
| 27 | 16.57 | C_15_H_1_0O_7_ | [M-H]^-^ | 2.395 | 301.03427, 273.04059,  178.99774, 151.00253 | Quercetin | Flavonoids |
| 28 | 18.03 | C_15_H_10_O_6_ | [M+H]^+^ | -2.942 | 287.05417, 241. 08472,  213. 05313, 165. 01765 | Citreorosein | Anthraquinones |
| 29 | 18.17 | C_15_H_10_O_6_ | [M-H]^-^ | 4.510 | 285.03936, 151.00261 | Luteolin | Flavonoids |
| 30 | 18.19 | C_17_H_14_O_7_ | [M-H]^-^ | 3.072 | 314.04343, 299.01968 | Jaceosidin | Flavonoids |
| 31 | 18.90 | C_12_H_8_O_4_ | [M+H]^+^ | -3.802 | 217.04871 | 5-Methoxypsoralen | Coumarins |
| 32 | 18.91 | C_16_H_10_O_6_ | [M-H]^-^ | 4.429 | 297.04068, 253.05057 | Rhein methylester | Anthraquinones |
| 33 | 19.47 | C_16_H_12_O_4_ | [M+H]^+^ | -2.250 | 254.05597, 213.08984,  137.02356 | Formononetin | Flavonoids |
| 34 | 19.69 | C_21_H_22_O_5_ | [M-H]^-^ | 1.270 | 119. 04897, 233. 08160,  353.13962, 218. 05791,  189. 09106, 165. 09119,  295. 06305 | Xanthohumol | Flavonoids |
| 35 | 19.93 | C_17_H_14_O_6_ | [M-H]^-^ | 4.234 | 313.07199, 298.04834, 283.02490, 255.02939 | Cirsimaritin | Flavonoids |
| 36 | 20.03 | C_20_H_20_O_5_ | [M-H]^-^ | 1.243 | 219. 06577, 339.12390,  119. 04896, 93. 03324,  175. 07561, 133. 06462,  151. 07518, 245. 08157,  233. 08165, 295. 13513,  193. 08624 | Sophoraflavanone B | Flavonoids |
| 37 | 20.37 | C_18_H_16_O_7_ | [M-H]^-^ | 2.247 | 343.08200, 328.05875, 313.03531 | Eupatilin | Flavonoids |
| 38 | 20.51 | C_25_H_30_O_7_ | [M-H]^-^ | 1.110 | 279. 16025, 161. 02335,  211. 16968, 305. 13977,  331. 15494, 237. 14967,  441.19189, 423. 18219 | Norkurarinol | Flavonoids |
| 39 | 20.59 | C_15_H_8_O_6_ | [M-H]^-^ | 4.507 | 283.02499, 239. 03467 | Rhein | Anthraquinones |
| 40 | 20.65 | C_26_H_30_O_7_ | [M-H]^-^ | 1.880 | 177. 01842, 275. 16553,  149. 02330, 139. 03902,  123. 04402, 421. 16553, | Kushenol N | Flavonoids |
| 41 | 21.06 | C_16_H_22_O_10_ | [M-H]^-^ | 3.003 | 373.12930, 149.00844 | Swertiamarin | Terpenes |
| 42 | 21.47 | C_26_H_32_O_7_ | [M-H]^-^ | 1.120 | 455.20755, 305. 13937,  331. 15530, 149. 05954,  123. 04387, 261. 15024,  314. 07898 | Kuraridinol | Flavonoids |
| 43 | 21.85 | C_25_H_28_O_7_ | [M-H]^-^ | 0.860 | 261. 14972, 177. 01846,  217. 05023, 421. 16632,  439.17599, 193. 15909,  137. 02324 | Kushenol X | Flavonoids |
| 44 | 22.02 | C_26_H_30_O_7_ | [M-H]^-^ | 2.270 | 453.19305, 177. 01593,  275. 16458, 303. 12390 | Kushenol I | Flavonoids |
| 45 | 22.03 | C_20_H_20_O_5_ | [M-H]^-^ | 1.200 | 193. 08624, 339.12390,  219. 06577 | Kushenol S | Flavonoids |
| 46 | 22.12 | C_25_H_30_O_6_ | [M-H]^-^ | 1.675 | 425.19754, 305. 13965,  331. 15509 | Kushenol T | Flavonoids |
| 47 | 22.11 | C_16_H_14_O_4_ | [M+H]^+^ | -3.008 | 271.09567 | Imperatorin | Coumarins |
| 48 | 22.34 | C_20_H_18_O_6_ | [M-H]^-^ | 1.275 | 353.10324, 219. 06567,  298. 04886, 175. 07561 | 8-Prenylkaempferol | Flavonoids |
| 49 | 22.43 | C_14_H_12_O_3_ | [M+H]^+^ | -4.107 | 229.08498, 214. 06152,  201. 12633 | Resveratrol | Stilbene |
| 50 | 22.78 | C_26_H_30_O_5_ | [M-H]^-^ | 2.020 | 161. 02335, 301. 14459,  421.20297 | Kushenol R | Flavonoids |
| 51 | 23.16 | C_27_H_32_O_6_ | [M-H]^-^ | 1.685 | 149. 05966, 301. 14453,  451.21320, 233. 15431,  201. 12807, 134. 03610, 367. 12170, 419. 18628, 315. 16116, 257. 15451,  163. 00246 | (2S)-2'-methoxykurarinone | Flavonoids |
| 52 | 23.54 | C_15_H_10_O_5_ | [M-H]^-^ | 4.721 | 269.04572, 241. 04973,  225. 05452 | Emodin | Anthraquinones |
| 53 | 23.67 | C_15_H_10_O_5_ | [M-H]^-^ | 4.572 | 269.04572, 241.04973, | Apigenin | Flavonoids |
| 54 | 23.68 | C_25_H_28_O_7_ | [M-H]^-^ | 0.960 | 261. 14944, 177. 01828,  217.15930, 287. 1308,  421. 16580, 439.17609, 124. 01515, 192. 07840,  149. 02318 | Kushenol L | Flavonoids |
| 55 | 23.73 | C_11_H_6_O_4_ | [M+H]^+^ | -2.833 | 203.03331, 159.04359,  147.04353, 131.04913 | Xanthotol | Coumarins |
| 56 | 23.75 | C_20_H_20_O_5_ | [M-H]^-^ | 1.200 | 339.12390, 193. 08632,  219. 06583, 321. 11301,  175. 07547, 265. 12527,  151. 07549, 133. 06458,  119. 04895 | Desmethylxanthohumol | Flavonoids |
| 57 | 24.68 | C_21_H_22_O_5_ | [M-H]^-^ | 1.300 | 119. 04890, 353.13965,  233. 08151, 218. 05809,  189. 09088, 165. 09111,  133. 06512, | Isoxanthohumol | Flavonoids |
| 58 | 24.74 | C_15_H_20_N_2_O | [M+H]^+^ | -4.119 | 245.16383, 176. 10594 | Sophoramine | Alkaloids |
| 59 | 24.74 | C_15_H_16_O_3_ | [M+H]^+^ | -1.309 | 245.11690, 189.05428,  131.04900, 103.05434 | Osthole | Coumarins |
| 60 | 25.55 | C_26_H_30_O_6_ | [M-H]^-^ | 1.305 | 161. 02327, 275. 16544,  151. 03885, 301. 14456,  419. 18735, 327. 16010,  191. 07138, 109. 02805 | Kurarinone | Flavonoids |
| 61 | 25.92 | C_26_H_30_O_6_ | [M-H]^-^ | 1.305 | 437.19717, 301. 14456, | Isokurarinone | Flavonoids |
| 62 | 28.28 | C_30_H_36_O_6_ | [M-H]^-^ | 3.215 | 329. 21249, 161. 02339,  205. 08638, 285. 22311,  355. 19324, 491.24603 | Kushenol B | Flavonoids |
